# From Chemical Drawing to Electronic Properties of Semiconducting Polymers in Bulk: A Tool for Chemical Discovery

**DOI:** 10.1021/acs.jctc.3c01417

**Published:** 2024-04-20

**Authors:** Colm Burke, Hesam Makki, Alessandro Troisi

**Affiliations:** Department of Chemistry and Materials Innovation Factory, University of Liverpool, Liverpool L69 7ZD, U.K.

## Abstract

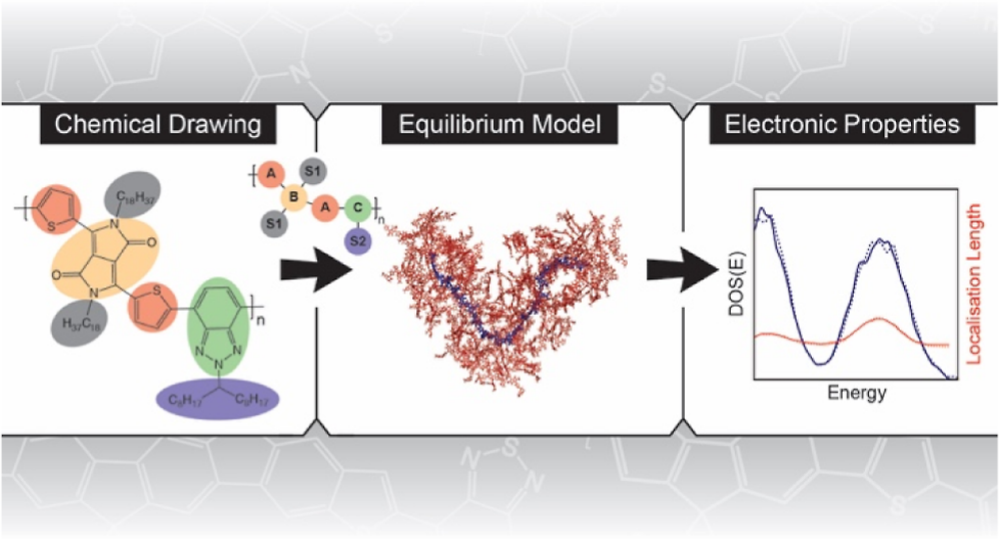

A quantum chemistry (QC)/molecular dynamics (MD) scheme is developed to calculate electronic properties of semiconducting polymers in three steps: (i) constructing the polymer force field through a unified workflow, (ii) equilibrating polymer models, and (iii) calculating electronic structure properties (e.g., density of states and localization length) from the equilibrated models by QC approaches. Notably, as the second step of this scheme is generally the most time-consuming one, we introduce an alternative method to compute thermally averaged electronic properties in bulk, based on the simulation of a polymer chain in the solution of its repeat units, which is shown to reproduce the microstructure of polymer chains and their electrostatic effect (successfully tested for five benchmark polymers) 10 times faster than state-of-the-art methods. In fact, this scheme offers a consistent and speedy way of estimating electronic properties of polymers from their chemical drawings, thus ensuring the availability of a homogeneous set of simulations to derive structure–property relationships and material design principles. As an example, we show how the electrostatic effect of the polymer chain environment can disturb the localized electronic states at the band tails and how this effect is more significant in the case of diketopyrrolopyrrole polymers as compared to indacenodithiophene and dithiopheneindenofluorene ones.

## 1 Introduction

Semiconducting polymers (SCPs) are one of the main classes of organic electronic materials able to display both high charge carrier mobility (up to 20 cm^2^ V^–1^ s^–1^) and excellent mechanical flexibility,^1,2^ making them a great candidate for flexible electronics.^3^ Moreover, the modular approach to their synthesis lends itself to a natural approach to molecular design,^4^ namely, the selection of a sequence of conjugated fragments and side chains in the repeat unit structure for a targeted application, e.g., organic photovoltaics,^5^ field effect transistors,^6^ light-emitting diodes,^7^ and bioelectronics,^8^ based on their optical, electronic, thermal, and mechanical properties.

The design rules, which are prerequisite for the molecular design, naturally emerge from structure–property data sets obtained from a homogeneous study (e.g., through using uniform force field parameters and constant level of theory and simulation setups) of many compounds. Computational studies of many SCPs can lead to structure–property relationships in the same way as typically carried out for small-molecule organic electronics.^9^ In this line, quantum chemistry (QC) methods have shown notable success in screening and discovery of small-molecule organic electronics based on desired electronic properties.^10^ For instance, they enabled the discovery of extremely rare organic electronic compounds,^11,12^ unravelled the effect of molecular chirality on their optoelectronic properties,^13^ and established (sometimes rather counterintuitive) design principles.^14^ Also, similar well-established QC methods are available to calculate electronic properties of SCPs, e.g., through the calculation of density of states (DOS)^15^ and by employing model reduction methods.^16^ Recent advances in the automation of such calculations show that a great insight into the design rules can be obtained by considering medium-to-large size data sets of SCPs.^17^ However, so far, high-throughput methods have been only reserved for single polymer chains in vacuum or implicit solvents,^18−21^ missing the (undeniable) role of intermolecular interactions on polymer conformation and the electrostatic effect of the surrounding polymer chains in bulk.

Molecular dynamics (MD) is an ideal method to construct high-quality SCP bulk models,^22−28^ from which one can include the intermolecular and electrostatic effects of the environment in electronic properties calculations through a hybrid QC/MD method.^15^ Such an approach consists of three main steps: (i) model construction, (ii) equilibration of the models, and (iii) QC calculations of the equilibrated models. The first step suffers from inconsistencies due to the various choices adopted for different force field parametrization methods, e.g., explicit, or implicit ways of determining the equilibrium value of bonded parameters or atomic charges, and is often a tedious and laborious step because of the many parameters such models require. The second step is generally the most time-consuming one, due to the long relaxation times of SCPs, which always scales unfavorably with the molecular weight.^29^ Also, there is no commonly accepted equilibration method due to the rather different microstructures of SCPs, e.g., from semicrystalline polymers to amorphous glasses, and the wide range of glass-transition temperature *T*_g_ (or melting points) for different SCPs.^30^ With respect to the third step, many alternatives are available including semiempirical,^31,32^ tight binding,^33,34^ and DFT methods,^15,35^ which reduce the comparability between different works. The aforementioned inhomogeneities and implemental difficulties resulted in studying a limited number of models, i.e., only a few high-quality polymer models per investigation have been possible.^36,37^ Thus, having fewer models studied and incomparable models generated in different studies has obstructed the development of structure–electronic property relationships required for formulating SCP design rules. Therefore, developing consistent and standardized SCP models, able to provide accurate electronic structures for a variety of polymers and a reasonable computational cost, will offer a great foundation for the application of the digital discovery approach to the class of SCPs.

In this paper, we put forward (i) a unified workflow to develop chemical-drawing-to-atomistic models for SCP chains and (ii) an accelerated approach to obtain electronic structure properties, which takes into account the intermolecular interactions and electrostatic environment of the surrounding chains. The accuracy of the QC/MD method is evaluated by comparing the morphological characteristics (e.g., torsion angle distribution, end-to-end distance, and radial distribution function) and electronic properties (e.g., DOS and DOS-driven properties), calculated for polymer chains sampled from (conventionally) equilibrated polymer melt models and those constructed by the faster alternative approach.

## 2 Method

### 2.1 Workflow to Develop Drawing-to-Atomistic Models

**Scheme 1:**
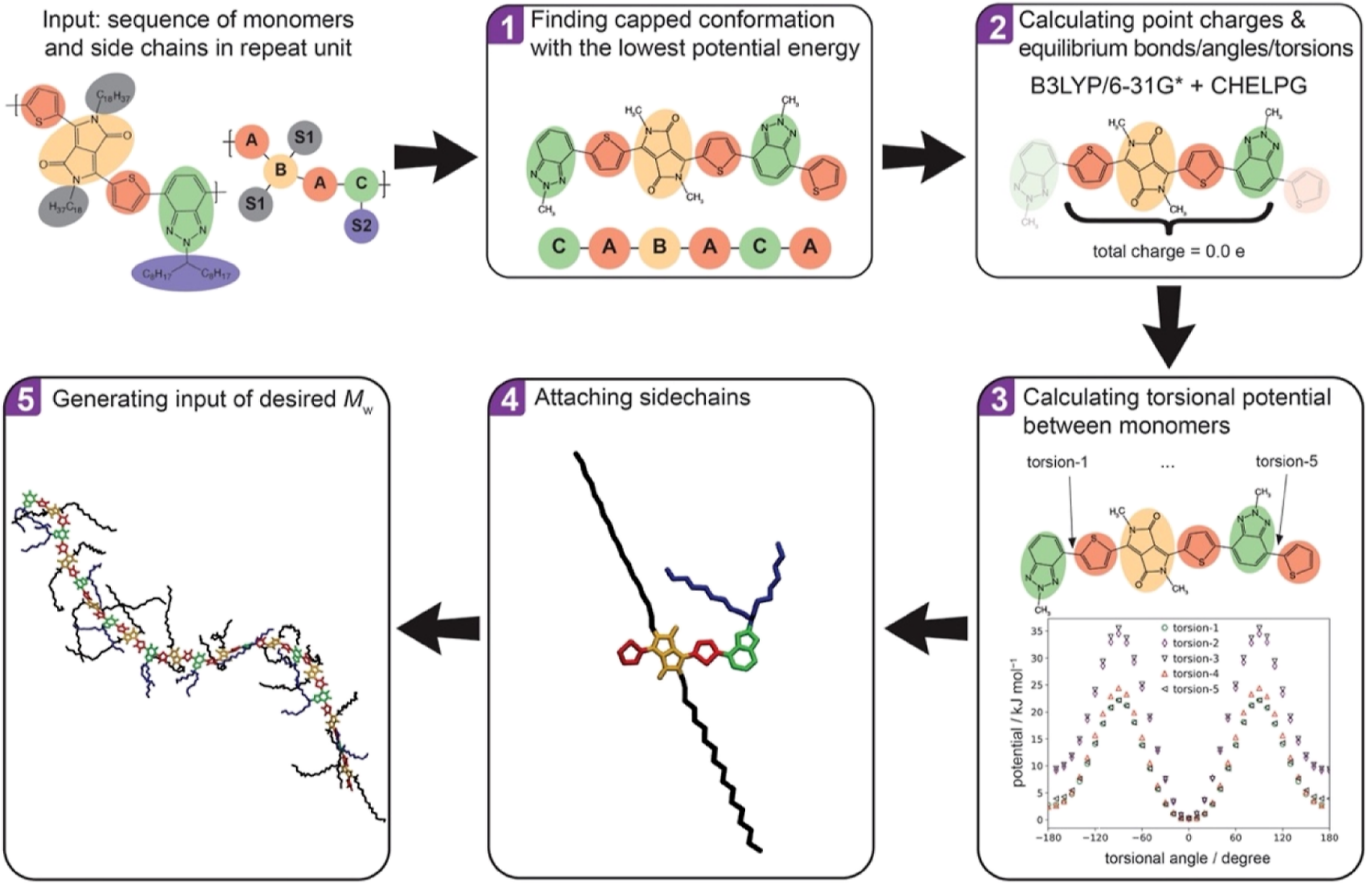
Workflow for Generating SCP Chain Models from Their Chemical Drawings

Scheme 1 shows a unified workflow for generating the SCP chain models. As shown, the conjugated monomers and their sequences in the polymer repeat unit are given as the input. One cannot assume that the net charge on each monomer is exactly zero (we have noticed up to |0.08|e shifting between monomers), i.e., the parameters of the individual monomer are not transferable, and the parametrization is best carried out using a larger model. We have considered here a minimal model where the repeat unit is capped with the first and last monomers and alkyl side-chains are replaced by methyl terminations (as shown in step 1 of Scheme 1). This model was used to compute the force field parameters including atomic point charges (we assume, as common in this area, that the point charges are not affected by the polymer conformation; see Supporting Information Section S1.1.2 for further validation of assumption) and bonded equilibrium parameters through DFT calculations (in this case B3LYP/6-31G*). The model of this size will also alleviate the possible dependency of the intermonomer torsional potential upon the neighboring monomers or the length of oligomer structure as observed before.^38^ We identified the lowest energy conformer for the model oligomer by an exhaustive search of all possible cis/trans configurations. Then, a universal DFT-based protocol is used to calculate the intra- and interfragment force field parameters (steps 2 and 3 in Scheme 1), see Supporting Information, Sections S1.1 and S1.2 for details. It should be noted that an important feature of this workflow is that each individual intrafragment bond, angle, and torsion angle equilibrium value is directly taken from the DFT-optimized minimal model, meaning that they are not only determined by the atom types forming the bond/angle/torsion, as is the case in generalized force fields such as OPLS-FF^39^ and GAFF,^40^ but the entire minimal model structure. This explicit method for bonded parameter calculation maintains the relative atomic positions (within each monomer) close to the real conjugated monomer structure during MD simulations. This feature is essential for the expected accuracy in calculating the electronic structures of SCP models (made by MD) due to the significance of relative atomic positions on the molecular orbital.^41−44^ Note that we used uniform bonded force constants for all conjugated monomers and assessed the implications of this assumption through a series of benchmark tests (see Supporting Information Section S1.2.1). The results demonstrated that this adjustment has a negligible effect on the distribution of bond lengths. The choice for DFT functional/basis set (in this version: B3LYP/6-31G*) was made to be consistent with the follow-up QC calculations which will be done directly on the MD snapshots, as explained in Section 2.3. Also, note that the workflow is designed to be adaptable, should different density functional or parent force field become desirable. The key point is that the protocol and choices are made once to be used consistently for all polymers modeled in one study.

After calculating the force field parameters for the minimal model (representing the backbone of the polymer), the side chains are attached to the designated positions, i.e., all methyl groups in the minimal polymer model, the side-chain force field parameters (united-atom and directly taken from OPLS-FF) are added, and the atomic charges around the side chain-backbone connection points are corrected to maintain the whole repeat-unit charge neutrality (step 4 in Scheme 1), see Supporting Information, Section S1.3. Last, polymer chain models (i.e., coordinates and force field files) with desired degrees of polymerization (e.g., in this study *n* = 10 since the effect of *M*_w_ on the calculated electronic structure properties was found to be negligible^27^ from *n* ≥ 10) are generated (step 5 in Scheme 1). Note that the Lennard-Jones parameters for all backbone atoms were also taken from the OPLS force field to be consistent with the side-chain parameters and the combination rule for the pairwise Lennard-Jones potential used in the force field is consistent with OPLS (i.e., geometric mean of the self-interaction parameters), see the formula in Supporting Information Section S1.1.1. It is worth emphasizing that a more accurate option for these parameters would be obtained through QM calculations, particularly for the conjugated fragments including heterocycles,^45^ which might give a more realistic microstructural property. Nevertheless, any choice can be made with respect to Lennard-Jones parameters as they are given to the workflow as part of the input files for any conjugated monomer.

### 2.2 Simplified Surrogate for MD Equilibration of the Models

**Figure 1:**
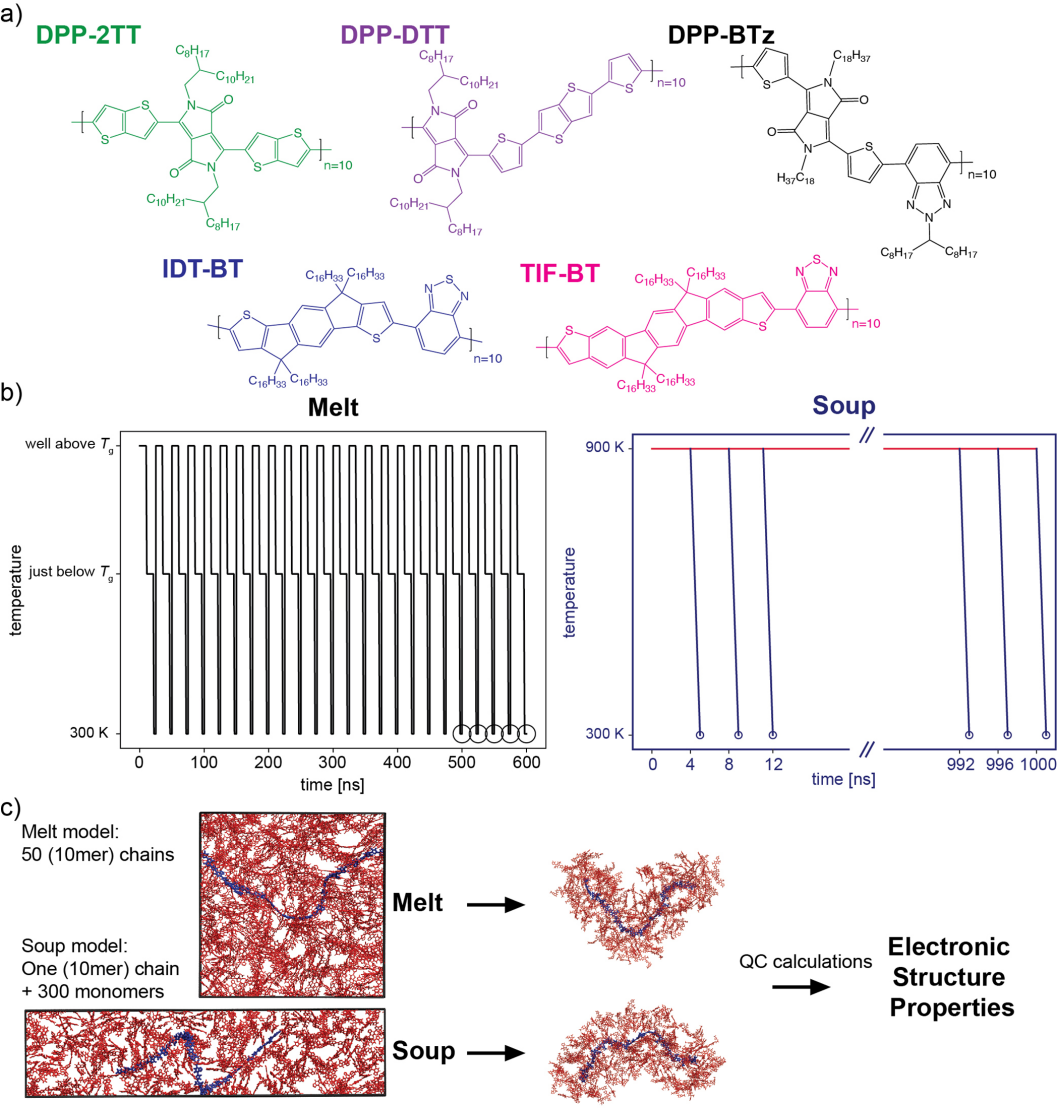
(a) Polymer structures. (b) Equilibration scheme for “melt” vs “soup” methods. Each large circle in the “melt” graph represents 50 chains sampled from one snapshot (therefore 50 × 5 = 250 samples in total), and each small circle in the “soup” graph represents one chain sampled from one snapshot (therefore, 1 × 250 = 250 samples in total). (c) Input generation from equilibrated structures by “melt” and “soup” methods for QC calculations. Side-chains are removed in the snapshots, (an example of) targeted chain for QC calculations are shown in blue, and the surrounding molecules are shown in red.

We used two methods for SCP model equilibration in this paper: (i) conventional method: a well-established equilibration protocol for SCPs (i.e., performing several annealing cycles, each includes above-*T*_g_/just-below-*T*_g_/room-temperature equilibration^26,27,46,47^), to which we refer to as “melt” for the rest of the manuscript, and (ii) a faster surrogate method: equilibrating one SCP chain in the soup of its repeat units at a temperature well above repeat unit melting point (i.e., 900 K, see Supporting Information, Section S2.1). This is an approximation of the ideal solvent for the chain, and we can hypothesize that it represents similar intermolecular interactions and electrostatic disorder. These hypotheses will be tested by comparison with the “melt” simulations for five different SCPs, as shown in Figure 1a (all of which are benchmark polymers belonging to the new generation of nonsemicrystalline SCP family). We will refer to this method as the “soup” for the rest of the manuscript. Examples of equilibrated simulation boxes for both “melt” (i.e., initially fully stretched 50 polymer chains in a cubic box) and “soup” (i.e., initially fully stretched one polymer chain and 300 repeat units in a rectangular box with an approximately 4:1:1 aspect ratio) methods are shown in Figure 1c. Note that in the case of “soup”, the polymer chain is initially aligned with the largest dimension of the rectangular box (i.e., *x*-axis) so that fewer pseudosolvent molecules (i.e., in our case, the repeat unit of the polymer) are needed compared to a cubic box. Furthermore, the repeat units surrounding the polymer chain in the “soup” approach represent the electrostatic effect of the surrounding polymer chains in “melt”; therefore, the atomic charges used to describe them are the same as the polymers’ repeat units. It is worth noting that by changing the number of pseudosolvents from 150 to 600, we noticed that from 200 molecules onward, the microstructural properties of the polymers (e.g., end-to-end distance) converge. Furthermore, as we discussed in Supporting Information, Section S1.5, the electrostatic effect of the surroundings cannot be captured by an implicit solvent method.

To equilibrate SCPs through the “melt” method, the *T*_g_ of the SCP model is essential for the simulation setting. Estimating *T*_g_ through MD simulations strongly depends on the calculation method^48^ due to the several factors, e.g., extremely high simulation cooling/heating rates, different fitting procedures, and considerably wide range of the transition region. In this work, we used two different methods to estimate the *T*_g_ of the SCP models, and we observe up to 300 K difference in the calculated values obtained from density–temperature and mean squared displacement (MSD)-temperature graphs, both from the same temperature-sweep simulation trajectories (see Supporting Information, Section S2.1). For our “melt” simulation settings, we used the values obtained from MSD curves as they give a direct estimation of the temperature at which a clear increase in the dynamics of polymer chains occurs. Thus, 1200 K was chosen to anneal all SCP models above their *T*_g_, and a temperature between 700 and 850 K (depending on the polymer type) for sub-*T*_g_ relaxations was used (see Supporting Information, Figure S11). This emphasizes another advantage of the “soup” method in which a constant high temperature (i.e., 900 K which is well above the melting points of all repeat units) can be used for any SCP, and the *T*_g_ estimation step (which is a prerequisite to the conventional “melt” equilibration) can be avoided.

For both “melt” and “soup” methods, after energy minimization, a rapid contraction of the box was done (under *NPT* and with *P* = 1000 bar) to quickly pack the box to the correct density. Equilibration simulations were performed under *NPT* conditions with a time step of 2 fs using GROMACS. A mass rescaling method for the backbone hydrogen atoms is employed to enable using larger time steps during MD equilibration.^49^ A 1.0 nm cutoff for Lennard-Jones and electrostatic interactions was used, all nonbonded interactions for 1–2 and 1–3 bonded pairs were excluded, and a scaling factor of 0.5 was used for 1–4 bonded pairs. The V-rescale thermostat and C-rescale barostat were used for packing steps, and the Nose–Hoover thermostat and Parrinello–Rahman barostat were employed for equilibration runs. The Verlet cutoff scheme was employed for nonbonded interactions, and Particle–mesh Ewald was used for long-range electrostatic interactions. Examples of coordinate, topology, and run files can be found in https://github.com/HMakkiMD/QCMD.

We previously verified the quality of the models generated by the workflow shown in Scheme 1 and equilibrated through the “melt” method for IDT-BT, where a strong agreement between the simulated X-ray scattering patterns from our models and the pattern given by GIWAXS measurement was achieved, see Figure 2 in ref (27). Similar analyses on the microstructures of two DPP-based (i.e., DPP-2TT and DPP-DTT) and two BT-based (i.e.. IDT-BT and TIF-BT) models obtained from “melt” equilibration have been done, and a comparison with experimental data is discussed in Section S2.2 of the Supporting Information, demonstrating that a single workflow can be successfully validated across a class of materials.

### 2.3 QC Calculations for a Chain in the “Shell” of Its Repeat Units

For each polymer, we sampled 250 chains, which are all statistically independent according to the block averaging analysis performed on SCP chain lengths, as shown and discussed in Supporting Information, Section S2.3. The electronic structure (orbital energies and localization) for each chain was computed taking into account their electrostatic environment (included via the point charges of all repeat units which have at least one atom within 2 nm distance from any atom in the polymer backbone) from both “melt” and “soup” simulation trajectories (see Figure 1c). These calculations are sped up with negligible loss of accuracy^50^ by using the smaller 3-21G* basis set and the same functional (B3LYP) used to derive the force field parameters. The DOS of each sample was calculated (see the QC calculation details in ref (15)) and averaged over 250 chains. It should be noted that in p-type organic semiconductors (including all the SCPs investigated in this paper), the shape of the DOS, particularly the distribution of states at the valence band edge, is closely tied to electronic disorder and hence charge mobility.^3,36,51^ Quantifying this, for example, via the slope of the valence band tail or by the charge carrier localization length (LL) at the valence band edge (see the Method in ref (15)) provides a valuable link between electronic structure calculation and experimentally measurable quantities, e.g., mobility.

Considering the smaller number of atoms in simulation box, the fast equilibration, and the exemption of *T*_g_ calculation for the “soup” method, obtaining 250 independent samples for QC calculations takes on average one-tenth of the computation time needed for the “melt” method. It should be noted that we used 20 annealing cycles for equilibration through the “melt” approach to cover a homogeneous setting for all polymers in this study (see Supporting Information, Section S2.2); however, the number of cycles needed for different polymers varies based on their relaxation time spectra and needed to be determined for each individual polymer by monitoring its properties during equilibration. This shows another advantage of the “soup” method, for which we use one simulation setting for any polymer at hand without monitoring its properties during equilibration. In Table 1, we summarize the computational cost, including the force field generation, MD equilibration, and QC calculations, for the “melt” and “soup” methods to achieve the same statistical sampling. In fact, this analysis shows that by employing the workflow for SCP model generation (as shown in Scheme 1), the calculation (including the investigator) time has been tremendously decreased (from typically a few months per polymer per investigator to below 2 weeks per polymer per computer node) so that the “melt” method is reasonably suitable to investigate electronic structure properties of any polymer of interest as well as several polymers in one investigation. Moreover, the “soup” method (with total calculation time of around 1–2 days per polymer per computer node) can be pushed to become a discovery tool where a large number (e.g., hundreds) of hypothetical models are explored in one study.

**Table 1:** Computational Time of “Melt” and “Soup” QC/MD Methods Considering the Five SCPs Discussed in This Paper^a^

	force field parametrization	MD equilibration	QC calculations	total
“melt” method	6–10 h	8–12 day	8–10 h	9–13 day
“soup” method	6–10 h	10–14 h	8–10 h	1–2 day

a Note that similar GPU- and CPU- computer nodes [one Nvidia A40 GPU/AMD EPYC 7443 CPU and one Intel(R) Xeon(R) Gold 6230, for MD and QC calculations, respectively] were used for all polymers through “melt” and “soup” methods.

All the scripts required to generate the starting configuration for “melt” and “soup” polymer simulation and its force field starting from the optimized structure of the monomers are freely available in https://github.com/HMakkiMD/GAMMPS. Additionally, in the repository, we have included input files required for simulating two example polymers mentioned in the paper (TIF-BT and DPP-BTz), as well as the output files that will be produced upon the successful execution of those scripts. We have made available the codes necessary for generating all input files for QC calculations from both “melt” and “soup” MD trajectories. Also, a module for calculating the density of states and localization length, along with expected output files for the two polymers, is also provided.

## 3 Results and Discussion

The electronic structure properties of SCPs are determined by the chain conformation and the electrostatic environment of the chain. Thus, the QC/MD method should accurately represent both characteristics to give reliable electronic properties. Therefore, in the first section, we analyze and compare the performance of “melt” and “soup” methods with respect to morphological properties, and in the second section, we evaluate their capabilities in predicting the electronic properties and reproducing the electrostatic environment.

### 3.1 Morphological Properties

**Figure 2:**
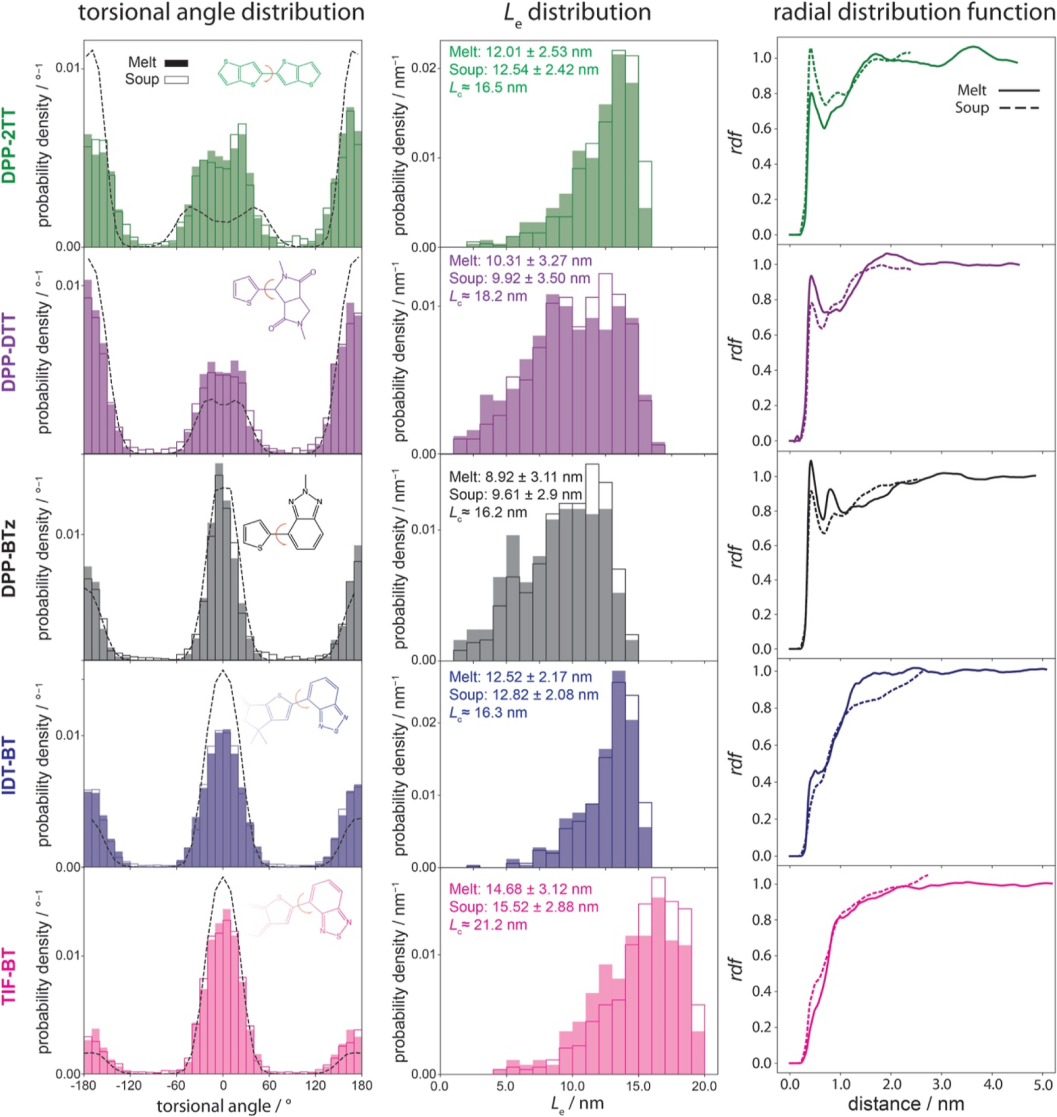
Torsional angle (left) and end-to-end distance *L*_e_ (middle) distributions for equilibrated polymer chains obtained from “melt”, filled bars and “soup”, unfilled bars, methods. The black dashed lines show the corresponding Boltzmann distribution of torsion angles as obtained from DFT scans on the representative repeat-unit molecule. The average and standard deviation from the average of *L*_e_ and contour length *L*_c_ of SCPs are given on each graph in the right panel. rdf (right) of all atoms in the backbone of a polymer chain as the “reference” and the other chains (for melt simulations)/repeat units (for soup simulations) as the “surrounding” atoms. Note that the intramolecule interactions are excluded in all rdf graphs.

The first microstructural analysis is the interfragment torsion distribution, i.e., one of the most significant parameters influencing electronic properties of SCPs,^52−54^ as obtained from “melt” and “soup” samples. Figure 2 (left panel) shows a comparison between the inter-repeat unit torsion distributions obtained from “melt” (filled bars) and “soup” (unfilled bars) samples, as well as the Boltzmann distribution (dashed line) calculated based on the input torsional potential (obtained from DFT scans on the repeat-unit representative molecule). As clearly shown in Figure 2 (left), the “soup” method can accurately capture the intermonomer torsion distribution for all polymers. Figure S15 in Supporting Information illustrates that a similar conclusion is valid for the other torsional potentials. Moreover, the deviation of torsion angle distributions obtained from MD simulations from the Boltzmann distribution quantifies the impact of surrounding molecules and side chains and the importance of using QC/MD schemes for calculating electronic structure properties instead of using ideal chain conformations for the QC calculations. It should be emphasized that the conformation of polymer chains in bulk is also influenced by (i) the mobility confinements imposed by the connectivity of the repeat units along the polymer chain, (ii) the conformation of side chains attached to the backbone, and (iii) the steric effect of surrounding molecules (i.e., polymers in case of “melt” and repeat units in case of “soup” models); thus, deviations in torsion angle distribution of MD models from the Boltzmann distribution are naturally expected. Also, such deviation is expected to be larger in the case of smaller torsion barriers; e.g., compare the deviation in φ_2TT-2TT_ (∼4*k*_B_T) torsion in the DPP-2TT polymer with φ_T-DPP_ (∼10*k*_B_T) in the DPP-DTT polymer in Figure 2.

The next important structural characteristic for SCPs is the chain end-to-end distance, *L*_e_, and its distribution. *L*_e_ quantifies how stretched polymer chains are in bulk, and it is an important property by which the robustness of the “soup” method in accurate representation of SCP microstructure can be evaluated. Figure 2 (middle panel) shows the average, standard deviation from the average, and distribution of *L*_e_ for all five polymers, as obtained from “melt” and “soup” samples. Note that an estimation of contour length *L*_c_ of polymers (i.e., the length of a fully stretched chain) is given on each graph. As shown, similar to the torsional distribution, the “soup” method generates very similar *L*_e_ and *L*_e_ distributions as compared to the “melt” method for all polymers. Note that the difference in the averages obtained from the two methods is well below the standard deviations obtained from “melt” simulations.

Radial distribution function (rdf) is another important analysis as it illustrates the intermolecular interactions of the models. Therefore, the rdf of all atoms within one polymer chain with respect to all other surrounding molecules (i.e., polymer chains in the case of “melt” and repeat units in the case of “soup”) are shown in Figure 2 (right panel). As shown, the most short-range interchain interactions have been captured by the “soup” method. However, the long-range orders cannot be reproduced by the “soup” method. This is, however, somehow expected due to the removal of the natural confinement imposed by the inter-repeat unit covalent bonds in surrounding polymer chains in case of “soup” models. We further investigated such differences by calculating the rdf of the center of mass of different monomers for “soup” and “melt” models in Supporting Information, Section S2.5. Nevertheless, as we will see in the next section, the difference in rdf between “soup” and “melt” snapshots does not necessarily translate into a difference in the main quantities of merit (DOS and LL).

We also calculated the ratio of monomers in the π–π interaction to the total number of monomers in the simulation boxes by using the methods explained in refs (26 and 27). Both “melt” and “soup” methods clearly show that DPP-based polymers contain a relatively higher number of π–π interactions; however, “soup” overestimates the pi-stacking pairs due to the easier movement/arrangement of repeat units compared to polymer chains (i.e., less bonded confinements), see Supporting Information Section S2.5. Thus, this feature clearly needs to be investigated via melt simulations.

### 3.2 Electronic Structure Properties

**Figure 3:**
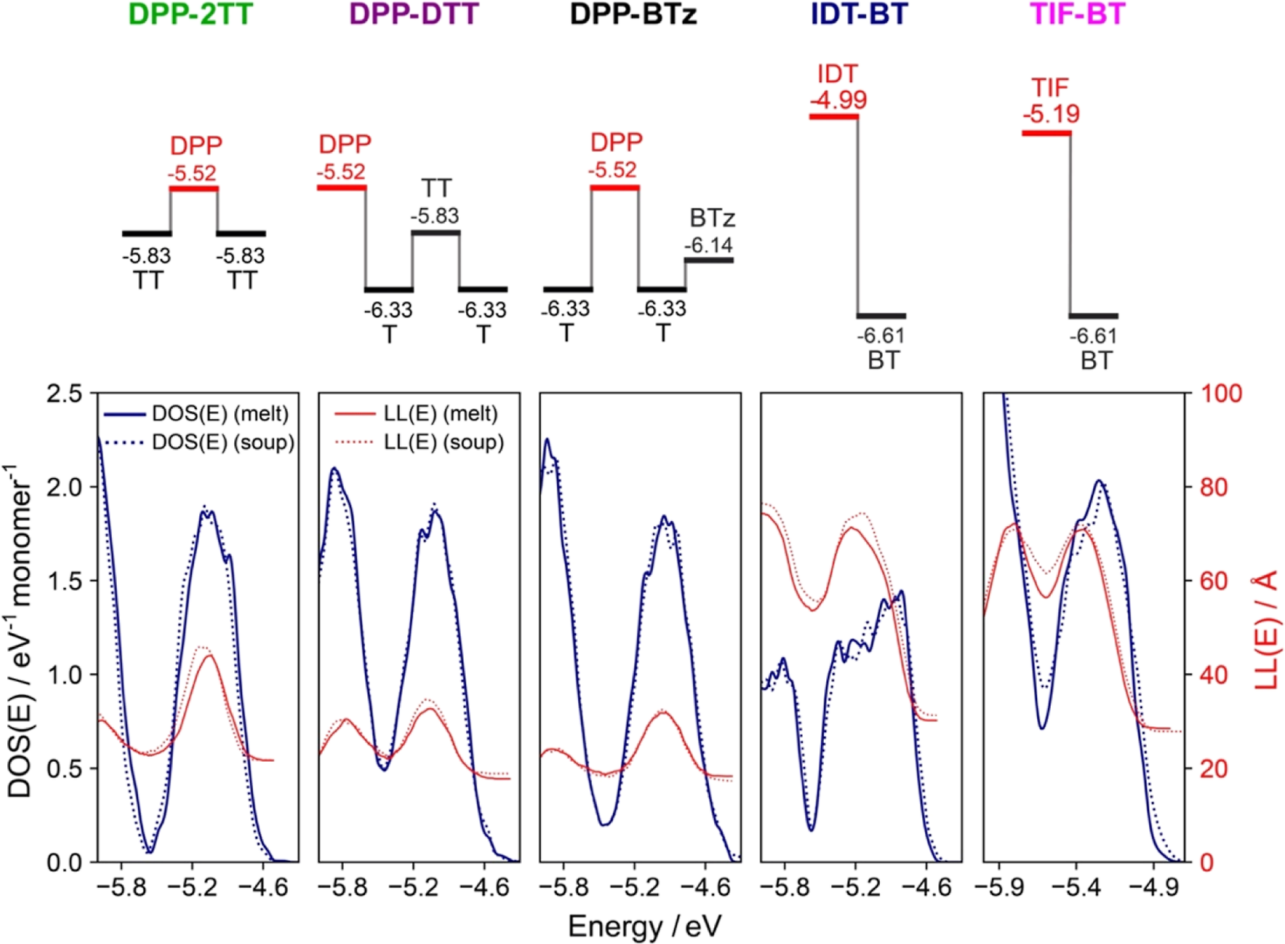
(Top) Abbreviated names and HOMO energies of monomers for each polymer repeat unit. Separation of horizontal bars indicates separation in energy. (Bottom) Density of states [DOS(E)] and localization lengths [LL(E)] for each polymer, averaged over 250 chain conformations. DOS and LL for “melt” and “soup” models are shown with solid and dotted lines, respectively.

Figure 3 shows the DOS and LL calculated based on “melt” (solid line) and “soup” (dashed line) models. The former property is recognized as determining charge carrier mobility in amorphous semiconductors, where charge transport is modeled by variable-range hopping between localized states,^55^ and the latter property (an orbital localization measure) is hence useful as a predictor of mobility in these materials. As can be seen, great agreement between the two calculations exists. This indicates that the two dominant factors determining electronic structure properties, i.e., chain conformation and electrostatic environment, can be accurately captured by the “soup” method, despite some clear differences observed in the interchain interactions between the “melt” and “soup” samples. Although the “soup” method is generally successful in replicating the properties calculated from “melt” models, the degree of agreement between the two differs slightly for different polymers. For instance, the “soup” method gives an almost identical prediction for the DOS and LL of DPP-based polymers modeled by the “melt” approach while for those containing BT as the acceptor, a slightly larger degree of mismatch can be seen. However, the difference between polymers is considerably larger than the difference between the two methods, which by definition identifies the “soup” as a predictive method. To clarify this point in a numerical way we calculated the band tail gradient and the energy at inflection point of the band tail for all polymers in the Supporting Information, Section S3.

**Figure 4:**
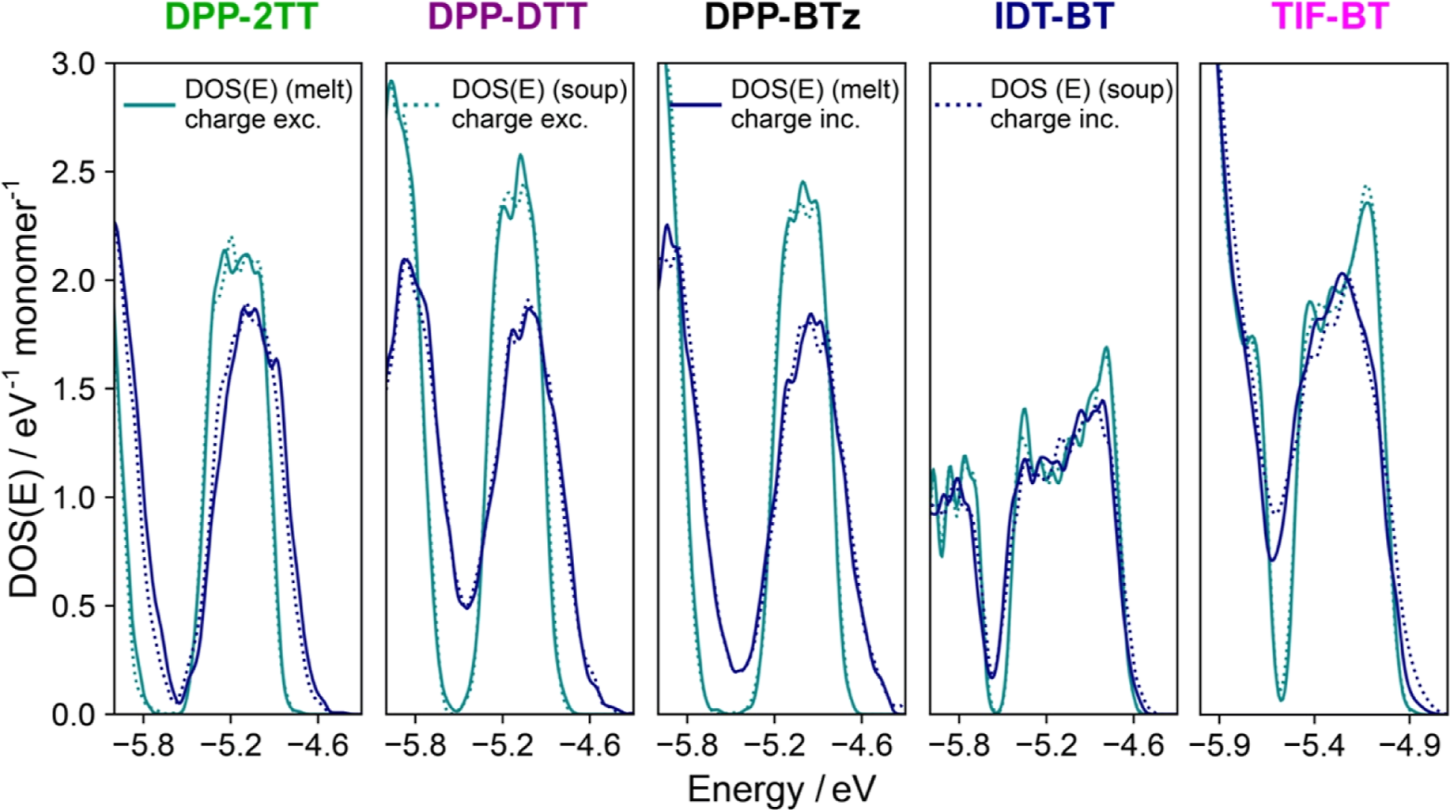
Energy-dependent density of states [DOS(E)] with/without electrostatic effects of the surrounding molecules, averaged over 250 chain conformations for each polymer.

As discussed in the Introduction section, the availability of a homogeneous set of simulations is helpful in extracting interesting structure–property relationships. For instance, these simulations allow the separation of the effects of the electrostatic disorder and conformational disorder on electronic structure properties of SCPs. Calculating the DOS of the five polymers in the absence of the surrounding charges (see Figure 4), we notice that the later have a very different impact on the DOS, more dramatic for DPP polymers and almost negligible for the IDT one so that the decrease in the slope of the DOS tail at the valence band edge due to the electrostatic disorder follows this order: DPP-BTz (2.20) > DPP-DTT (2.06) > DPP-2TT = TIF-BT (1.51) > IDT-BT (1.29 eV^–2^ monomer^–1^).

This can be rationalized by looking at the variation of the electrostatic potential (EP) generated by these polymers around each chain. We have evaluated them through computing the EP disorder generated by the atoms in the repeat unit structure on a shell surrounding the repeat unit (Supporting Information, Section S3). The distribution of EPs around DPP-based polymers (standard deviation of EPs is ∼0.60 V) is relatively wider as compared to IDT- and TIF-based ones (∼0.40 V). Moreover, we know that the decrease in the DOS tail slope due to the environment correlates with the increase in electrostatic disorder felt by the highest HOMO monomers in each polymer (i.e., DPP, TIF, and IDT) from the surrounding molecules. To this end, we calculated the EP exerted by the surrounding molecules on a shell around DPP, TIF, and IDT monomers for DPP-BTz, TIF-BT, and IDT-BT polymers. As expected, DPP feels the largest electrostatic disorder (0.319 V), followed by TIF (0.177 V) and IDT (0.142 V), most likely due to (i) the larger disturbance imposed by the surrounding chains and (ii) the smaller size of DPP compared to IDT and TIF. Note that the EP disorder projected onto the center of mass of the monomers show a similar trend, i.e., 0.21, 0.12, and 0.10 V for DPP-BTz, TIF-BT, and IDT-BT, respectively. Therefore, such calculations can constitute an easy design principle to consider for the development of new materials. This is an example of how structure–property relationships are more easily derived by comparing across consistent sets of simulations.

## 4 Conclusions

In this paper, we put forward a workflow to obtain atomistic models from SCP chemical drawings. We suggested a simplified alternative equilibration method (i.e., “soup” method) which can accurately predict intramolecular conformations and electronic properties (density of states and localization length) of a range of polymers. More importantly, it enables an accurate estimation of SCP electronic properties with a constant calculation setup (e.g., simulation box dimension, simulation time, temperature, and equilibration scheme), regardless of the polymer structure. Our QC/MD scheme tremendously reduces the calculation time from typically a few months per polymer (through manual and conventional methods) to a few days per polymer. While the “soup” approach is suitable for rapid screening (i.e., 1–2 days per polymer per computer node) of electronic properties for many SCPs without taking into account the interchain transport properties, the “melt” approach provides detailed intra- and interchain properties, within a reasonable time frame (i.e., 1–2 weeks per polymer per computer node). Therefore, the two proposed methods tremendously facilitate future endeavors in exploring a large space of SCPs with directly comparable methods by providing a screening funnel consists of two levels of calculations: (i) the “soup” method for the first layer and speedy screening based on intrachain transport properties and (ii) the “melt” method for advanced analyses of microstructural and electronic properties of the screened polymers. Therefore, this paper presents a framework that has the potential to significantly expand the number of polymers under investigation and the electronic properties explored in future studies. Last, we show that the availability of a homogeneous set of simulations is helpful in extracting interesting structure–property relationships, in this case: the greater effect of the surrounding polymer chains on the DOS of DPP polymers in comparison to the others.

## Supporting Information Available

Force field parametrization and model details; equilibration and analyses details; and electrostatic disorder generated by SCP on the surroundings (PDF)

## References

[ref1] Kim M.; Ryu S. U.; Park S. A.; Choi K.; Kim T.; Chung D.; Park T. Donor-Acceptor-Conjugated Polymer for High-Performance Organic Field-Effect Transistors: A Progress Report. Adv. Funct. Mater. 2020, 30 (20), 1904545 10.1002/adfm.201904545.

[ref2] Paterson A. F.; Singh S.; Fallon K. J.; Hodsden T.; Han Y.; Schroeder B. C.; Bronstein H.; Heeney M.; McCulloch I.; Anthopoulos T. D. Recent Progress in High-Mobility Organic Transistors: A Reality Check. Adv. Mater. 2018, 30 (36), 1801079 10.1002/adma.201801079. 30022536

[ref3] Wang M.; Baek P.; Akbarinejad A.; Barker D.; Travas-Sejdic J. Conjugated Polymers and Composites for Stretchable Organic Electronics. J. Mater. Chem. C 2019, 7 (19), 5534–5552. 10.1039/C9TC00709A.

[ref4] Ding L.; Yu Z. Di; Wang X. Y.; Yao Z. F.; Lu Y.; Yang C. Y.; Wang J. Y.; Pei J. Polymer Semiconductors: Synthesis, Processing, and Applications. Chem. Rev. 2023, 123 (12), 7421–7497. 10.1021/acs.chemrev.2c00696. 37232480

[ref5] Dang D.; Yu D.; Wang E. Conjugated Donor-Acceptor Terpolymers Toward High-Efficiency Polymer Solar Cells. Adv. Mater. 2019, 31 (22), 1807019 10.1002/adma.201807019. 30701605

[ref6] Holliday S.; Donaghey J. E.; McCulloch I. Advances in Charge Carrier Mobilities of Semiconducting Polymers Used in Organic Transistors. Chem. Mater. 2014, 26 (1), 647–663. 10.1021/cm402421p.

[ref7] Ostroverkhova O. Organic Optoelectronic Materials: Mechanisms and Applications. Chem. Rev. 2016, 116 (22), 13279–13412. 10.1021/acs.chemrev.6b00127. 27723323

[ref8] Dimov I. B.; Moser M.; Malliaras G. G.; McCulloch I. Semiconducting Polymers for Neural Applications. Chem. Rev. 2022, 122 (4), 4356–4396. 10.1021/acs.chemrev.1c00685. 35089012 PMC9007464

[ref9] Bronstein H.; Nielsen C. B.; Schroeder B. C.; McCulloch I. The Role of Chemical Design in the Performance of Organic Semiconductors. Nat. Rev. Chem 2020, 4 (2), 66–77. 10.1038/s41570-019-0152-9. 37128048

[ref10] Omar Ö. H.; Del Cueto M.; Nematiaram T.; Troisi A. High-Throughput Virtual Screening for Organic Electronics: A Comparative Study of Alternative Strategies. J. Mater. Chem. C 2021, 9 (39), 13557–13583. 10.1039/D1TC03256A. PMC8515942 34745630

[ref11] Terence Blaskovits J.; Garner M. H.; Corminboeuf C. Symmetry-Induced Singlet-Triplet Inversions in Non-Alternant Hydrocarbons. Angew. Chem., Int. Ed. 2023, 62 (15), e202218156 10.1002/anie.202218156. 36786076

[ref12] Omar Ö. H.; Xie X.; Troisi A.; Padula D. Identification of Unknown Inverted Singlet-Triplet Cores by High-Throughput Virtual Screening. J. Am. Chem. Soc. 2023, 145 (36), 19790–19799. 10.1021/jacs.3c05452. 37639703 PMC10510316

[ref13] Yang Y.; Rice B.; Shi X.; Brandt J. R.; Correa Da Costa R.; Hedley G. J.; Smilgies D. M.; Frost J. M.; Samuel I. D. W.; Otero-De-La-Roza A.; Johnson E. R.; Jelfs K. E.; Nelson J.; Campbell A. J.; Fuchter M. J. Emergent Properties of an Organic Semiconductor Driven by Its Molecular Chirality. ACS Nano 2017, 11 (8), 8329–8338. 10.1021/acsnano.7b03540. 28696680

[ref14] Nematiaram T.; Troisi A. Strategies to Reduce the Dynamic Disorder in Molecular Semiconductors. Mater. Horiz. 2020, 7 (11), 2922–2928. 10.1039/D0MH01159B.

[ref15] Qin T.; Troisi A. Relation between Structure and Electronic Properties of Amorphous MEH-PPV Polymers. J. Am. Chem. Soc. 2013, 135 (30), 11247–11256. 10.1021/ja404385y. 23829780

[ref16] Prodhan S.; Manurung R.; Troisi A. From Monomer Sequence to Charge Mobility in Semiconductor Polymers via Model Reduction. Adv. Funct. Mater. 2023, 33 (36), 1–10. 10.1002/adfm.202303234.

[ref17] Manurung R.; Troisi A. Screening Semiconducting Polymers to Discover Design Principles for Tuning Charge Carrier Mobility. J. Mater. Chem. C 2022, 10 (38), 14319–14333. 10.1039/D2TC02527B. PMC9536249 36325475

[ref18] Jackson N. E.; Kohlstedt K. L.; Savoie B. M.; Olvera De La Cruz M.; Schatz G. C.; Chen L. X.; Ratner M. A. Conformational Order in Aggregates of Conjugated Polymers. J. Am. Chem. Soc. 2015, 137 (19), 6254–6262. 10.1021/jacs.5b00493. 25920989

[ref19] Jackson N. E.; Savoie B. M.; Kohlstedt K. L.; Marks T. J.; Chen L. X.; Ratner M. A. Structural and Conformational Dispersion in the Rational Design of Conjugated Polymers. Macromolecules 2014, 47 (3), 987–992. 10.1021/ma4023923.

[ref20] Wilbraham L.; Berardo E.; Turcani L.; Jelfs K. E.; Zwijnenburg M. A. High-Throughput Screening Approach for the Optoelectronic Properties of Conjugated Polymers. J. Chem. Inf. Model. 2018, 58 (12), 2450–2459. 10.1021/acs.jcim.8b00256. 29940733 PMC6307085

[ref21] Manurung R.; Li P.; Troisi A. Rapid Method for Calculating the Conformationally Averaged Electronic Structure of Conjugated Polymers. J. Phys. Chem. B 2021, 125 (23), 6338–6348. 10.1021/acs.jpcb.1c02866. 34097424

[ref22] Melnyk A.; Junk M. J. N.; McGehee M. D.; Chmelka B. F.; Hansen M. R.; Andrienko D. Macroscopic Structural Compositions of π-Conjugated Polymers: Combined Insights from Solid-State NMR and Molecular Dynamics Simulations. J. Phys. Chem. Lett. 2017, 8 (17), 4155–4160. 10.1021/acs.jpclett.7b01443. 28809493

[ref23] Matta M.; Wu R.; Paulsen B. D.; Petty A. J.; Sheelamanthula R.; McCulloch I.; Schatz G. C.; Rivnay J. Ion Coordination and Chelation in a Glycolated Polymer Semiconductor: Molecular Dynamics and X-Ray Fluorescence Study. Chem. Mater. 2020, 32 (17), 7301–7308. 10.1021/acs.chemmater.0c01984.

[ref24] Moro S.; Siemons N.; Drury O.; Warr D. A.; Moriarty T. A.; Perdigão L. M. A.; Pearce D.; Moser M.; Hallani R. K.; Parker J.; McCulloch I.; Frost J. M.; Nelson J.; Costantini G. The Effect of Glycol Side Chains on the Assembly and Microstructure of Conjugated Polymers. ACS Nano 2022, 16 (12), 21303–21314. 10.1021/acsnano.2c09464. 36516000 PMC9798861

[ref25] Roscioni O. M.; Ricci M.; Zannoni C.; D’Avino G. Are Coarse-Grained Structures as Good as Atomistic Ones for Calculating the Electronic Properties of Organic Semiconductors?. J. Phys. Chem. C 2023, 127 (19), 9225–9235. 10.1021/acs.jpcc.2c08862.

[ref26] Makki H.; Troisi A. Morphology of Conducting Polymer Blends at the Interface of Conducting and Insulating Phases: Insight from PEDOT:PSS Atomistic Simulations. J. Mater. Chem. C 2022, 10 (42), 16126–16137. 10.1039/D2TC03158B. PMC9632246 36387833

[ref27] Makki H.; Burke C. A.; Troisi A. Microstructural Model of Indacenodithiophene-Co-Benzothiadiazole Polymer: Π-Crossing Interactions and Their Potential Impact on Charge Transport. J. Phys. Chem. Lett. 2023, 14, 8867–8873. 10.1021/acs.jpclett.3c02305. 37756473 PMC10561260

[ref28] Venkateshvaran D.; Nikolka M.; Sadhanala A.; Lemaur V.; Zelazny M.; Kepa M.; Hurhangee M.; Kronemeijer A. J.; Pecunia V.; Nasrallah I.; Romanov I.; Broch K.; McCulloch I.; Emin D.; Olivier Y.; Cornil J.; Beljonne D.; Sirringhaus H. Approaching Disorder-Free Transport in High-Mobility Conjugated Polymers. Nature 2014, 515 (7527), 384–388. 10.1038/nature13854. 25383522

[ref29] Jackson J. K.; De Rosa M. E.; Winter H. Molecular Weight Dependence of Relaxation Time Spectra for the Entanglement and Flow Behavior of Monodisperse Linear Flexible Polymers. Macromolecules 1994, 27, 2426–2431. 10.1021/ma00087a010.

[ref30] Botiz I.; Durbin M. M.; Stingelin N. Providing a Window into the Phase Behavior of Semiconducting Polymers. Macromolecules 2021, 54 (12), 5304–5320. 10.1021/acs.macromol.1c00296.

[ref31] Cheung D. L.; McMahon D. P.; Troisi A. A Realistic Description of the Charge Carrier Wave Function in Microcrystalline Polymer Semiconductors. J. Am. Chem. Soc. 2009, 131 (31), 11179–11186. 10.1021/ja903843c. 19621930

[ref32] D’Avino G.; Mothy S.; Muccioli L.; Zannoni C.; Wang L.; Cornil J.; Beljonne D.; Castet F. Energetics of Electron–Hole Separation at P3HT/PCBM Heterojunctions. J. Phys. Chem. C 2013, 117, 12981–12990. 10.1021/jp402957g.

[ref33] Heck A.; Kranz J. J.; Elstner M. Simulation of Temperature-Dependent Charge Transport in Organic Semiconductors with Various Degrees of Disorder. J. Chem. Theory Comput. 2016, 12 (7), 3087–3096. 10.1021/acs.jctc.6b00215. 27224054

[ref34] McMahon D. P.; Troisi A. An Ad Hoc Tight Binding Method to Study the Electronic Structure of Semiconducting Polymers. Chem. Phys. Lett. 2009, 480 (4–6), 210–214. 10.1016/j.cplett.2009.09.032.

[ref35] Schütze Y.; Gayen D.; Palczynski K.; de Oliveira Silva R.; Lu Y.; Tovar M.; Partovi-Azar P.; Bande A.; Dzubiella J. How Regiochemistry Influences Aggregation Behavior and Charge Transport in Conjugated Organosulfur Polymer Cathodes for Lithium-Sulfur Batteries. ACS Nano 2023, 17 (8), 7889–7900. 10.1021/acsnano.3c01523. 37014093 PMC10141565

[ref36] Siemons N.; Pearce D.; Yu H.; Tuladhar S. M.; LeCroy G. S.; Sheelamanthula R.; Hallani R. K.; Salleo A.; McCulloch I.; Giovannitti A.; Frost J. M.; Nelson J. Controlling swelling in mixed transport polymers through alkyl side-chain physical cross-linking. Proc. Natl. Acad. Sci. U.S.A. 2023, 120 (35), e2306272120 10.1073/pnas.2306272120. 37603750 PMC10467570

[ref37] Lemaur V.; Cornil J.; Lazzaroni R.; Sirringhaus H.; Beljonne D.; Olivier Y. Resilience to Conformational Fluctuations Controls Energetic Disorder in Conjugated Polymer Materials: Insights from Atomistic Simulations. Chem. Mater. 2019, 31 (17), 6889–6899. 10.1021/acs.chemmater.9b01286.

[ref38] Sears J. S.; Chance R. R.; Brédas J. L. Torsion Potential in Polydiacetylene: Accurate Computations on Oligomers Extrapolated to the Polymer Limit. J. Am. Chem. Soc. 2010, 132 (38), 13313–13319. 10.1021/ja103769j. 20825183

[ref39] Jorgensen W. L.; Maxwell D. S.; Tirado-Rives J. Development and Testing of the OPLS All-Atom Force Field on Conformational Energetics and Properties of Organic Liquids. J. Am. Chem. Soc. 1996, 118 (45), 11225–11236. 10.1021/ja9621760.

[ref40] Wang J.; Wolf R. M.; Caldwell J. W.; Kollman P. A.; Case D. A. Development and Testing of a General Amber Force Field. J. Comput. Chem. 2004, 25 (9), 1157–1174. 10.1002/jcc.20035. 15116359

[ref41] Cheung D. L.; Mcmahon D. P.; Troisi A. Computational Study of the Structure and Charge-Transfer Parameters in Low-Molecular-Mass P3HT. J. Phys. Chem. B 2009, 113 (28), 9393–9401. 10.1021/jp904057m. 19537781

[ref42] Cacelli I.; Prampolini G. Parametrization and Validation of Intramolecular Force Fields Derived from DFT Calculations. J. Chem. Theory Comput. 2007, 3 (5), 1803–1817. 10.1021/ct700113h. 26627623

[ref43] Grimme S. A General Quantum Mechanically Derived Force Field (QMDFF) for Molecules and Condensed Phase Simulations. J. Chem. Theory Comput. 2014, 10 (10), 4497–4514. 10.1021/ct500573f. 26588146

[ref44] Claridge K.; Troisi A. Developing Consistent Molecular Dynamics Force Fields for Biological Chromophores via Force Matching. J. Phys. Chem. B 2019, 123 (2), 428–438. 10.1021/acs.jpcb.8b10746. 30565460

[ref45] Jacobs M.; Greff Da Silveira L.; Prampolini G.; Livotto P. R.; Cacelli I. Interaction Energy Landscapes of Aromatic Heterocycles through a Reliable yet Affordable Computational Approach. J. Chem. Theory Comput. 2018, 14 (2), 543–556. 10.1021/acs.jctc.7b00602. 29300481

[ref46] Michaels W.; Zhao Y.; Qin J. Atomistic Modeling of PEDOT:PSS Complexes II: Force Field Parameterization. Macromolecules 2021, 54 (12), 5354–5365. 10.1021/acs.macromol.1c00860.

[ref47] Keene S. T.; Michaels W.; Melianas A.; Quill T. J.; Fuller E. J.; Giovannitti A.; McCulloch I.; Talin A. A.; Tassone C. J.; Qin J.; Troisi A.; Salleo A. Efficient Electronic Tunneling Governs Transport in Conducting Polymer-Insulator Blends. J. Am. Chem. Soc. 2022, 144 (23), 10368–10376. 10.1021/jacs.2c02139. 35658455 PMC9204759

[ref48] Patrone P. N.; Dienstfrey A.; Browning A. R.; Tucker S.; Christensen S. Uncertainty Quantification in Molecular Dynamics Studies of the Glass Transition Temperature. Polymer 2016, 87, 246–259. 10.1016/j.polymer.2016.01.074.

[ref49] Hopkins C. W.; Le Grand S.; Walker R. C.; Roitberg A. E. Long-Time-Step Molecular Dynamics through Hydrogen Mass Repartitioning. J. Chem. Theory Comput. 2015, 11 (4), 1864–1874. 10.1021/ct5010406. 26574392

[ref50] Omar Ö. H.; Nematiaram T.; Troisi A.; Padula D. Organic Materials Repurposing, a Data Set for Theoretical Predictions of New Applications for Existing Compounds. Sci. Data 2022, 9 (1), 54 10.1038/s41597-022-01142-7. 35165288 PMC8844419

[ref51] Thomas T. H.; Harkin D. J.; Gillett A. J.; Lemaur V.; Nikolka M.; Sadhanala A.; Richter J. M.; Armitage J.; Chen H.; McCulloch I.; Menke S. M.; Olivier Y.; Beljonne D.; Sirringhaus H. Short Contacts between Chains Enhancing Luminescence Quantum Yields and Carrier Mobilities in Conjugated Copolymers. Nat. Commun. 2019, 10 (1), 2614 10.1038/s41467-019-10277-y. 31197152 PMC6565747

[ref52] Yan X.; Xiong M.; Deng X. Y.; Liu K. K.; Li J. T.; Wang X. Q.; Zhang S.; Prine N.; Zhang Z.; Huang W.; Wang Y.; Wang J. Y.; Gu X.; So S. K.; Zhu J.; Lei T. Approaching Disorder-Tolerant Semiconducting Polymers. Nat. Commun. 2021, 12 (1), 5723 10.1038/s41467-021-26043-y. 34588457 PMC8481336

[ref53] Kanimozhi C.; Naik M.; Yaacobi-Gross N.; Burnett E. K.; Briseno A. L.; Anthopoulos T. D.; Patil S. Controlling Conformations of Diketopyrrolopyrrole-Based Conjugated Polymers: Role of Torsional Angle. J. Phys. Chem. C 2014, 118 (22), 11536–11544. 10.1021/jp501526h.

[ref54] Darling S. B. Isolating the Effect of Torsional Defects on Mobility and Band Gap in Conjugated Polymers. J. Phys. Chem. B 2008, 112 (30), 8891–8895. 10.1021/jp8017919. 18597518

[ref55] Tessler N.; Preezant Y.; Rappaport N.; Roichman Y. Charge Transport in Disordered Organic Materials and Its Relevance to Thin-Film Devices: A Tutorial Review. Adv. Mater. 2009, 21 (27), 2741–2761. 10.1002/adma.200803541.

