# Supplementary material for: From Chemical Drawing to Electronic Properties of Semiconducting Polymers in Bulk: A Tool for Chemical Discovery
Source: J Chem Theory Comput. 2024 Apr 20;20(9):4019–28. doi: 10.1021/acs.jctc.3c01417 (PMC11099970; doi:10.1021/acs.jctc.3c01417)
Supplement: Supplementary file 1 — ct3c01417_si_001.pdf [file ct3c01417_si_001.pdf]

Supporting Information For:

From Chemical Drawing to Electronic Properties of Semiconducting Polymers in Bulk: A Tool for Chemical Discovery

Colm Burke[1], Hesam Makki[1], Alessandro Troisi[1]*

[1]Department of Chemistry and Materials Innovation Factory, University of Liverpool, Liverpool L69 7ZD, U.K.

Corresponding Author:
Hesam Makki
Email: h.makki@liverpool.ac.uk

S1. Force field parametrisation and model details

Force field parameters of the backbone and sidechains of the polymer were obtained separately. OPLS force field was used for bonded and non-bonded parameters of the side chains. The backbone parameters were derived from the optimised structure of the representative molecule for the polymer repeat unit (see Figure S1), in which the repeat unit structure (e.g., A-B-C) is capped with the first and last fragments (e.g., C-A-B-C-A). Also, each sidechain is represented by a methyl group. Note that all DFT calculations were performed by Gaussian 16 by using B3lyp hybrid functional and 6-31G* basis set for all steps of force field calculations.

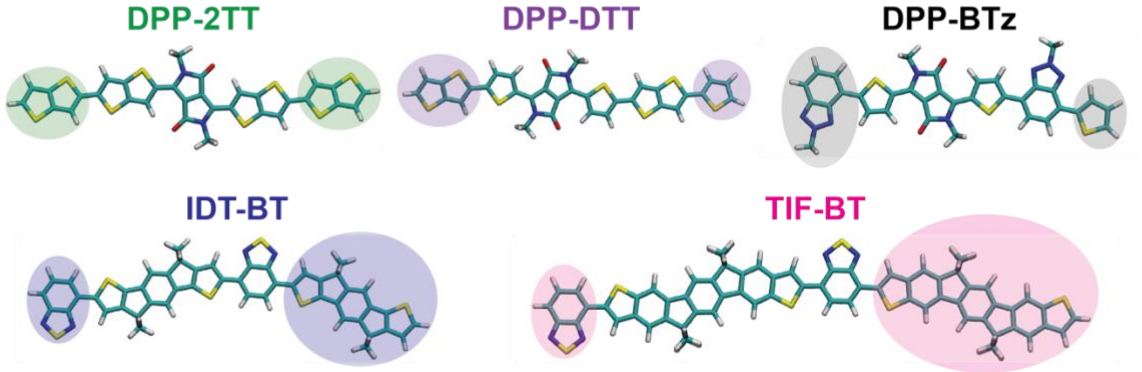

Figure S1. Representative molecule for polymer repeat unit. The capping fragments are shaded, and C, S, N, O, and H atoms are shown in cyan, yellow, blue, red, and white colours.

The backbone non-bonded and bonded parameterisation methods are detailed in “non-bonded parameters” and “bonded parameters”, respectively. The sidechain attachment procedure and the backbone-sidechain connection point parametrisation are explained in the section “sidechain attachment procedure”. “Polymer models” summarises the structure of the resulting polymers.

S1.1. Non-bonded parameters

S1.1.1. Lennard Jones parameters

The Lennard jones parameters of atoms in the polymer backbone were directly taken from their analogues in OPLS force field. The pairwise LJ potentials (for intra- and inter-molecular interactions) are calculated by $C_{ij}^6 = (C_i^6\ C_j^6)^{0.5}$ and $C_{ij}^{12} = (C_i^{12}\ C_j^{12})^{0.5}$. The LJ and coulombic interactions are considered for all atoms in the simulation (including conjugated backbones and sidechains); however, these interactions for atoms that are no further than 3 bonds away are excluded and for 1-4 interactions are multiplied by 0.5.

S1.1.2. Atomic charge of backbone (conjugated part)

The atomic charges were calculated by using CHELPG scheme (developed by Breneman and Wiberg, J. Comp. Chem. 1990, 11, 361) on the optimised molecule with the lowest potential energy. An example of resulting point charges for IDT-BT from CHELPG computation for one repeat unit (RU, i.e., the representative molecule for polymer repeat unit after removing the capping fragments, see Figure S2) are shown in table S1. In this case, the sum of point charges for the whole monomer was zero and, after removing the capping BT and IDT fragments, the total charge of one RU was calculated –0.004 e. The extra charge of the RU was redistributed on all atoms (equally) so that the total charge of each RU in the polymers equals to zero. Note that the total charge on capping fragments for all polymers never exceeded ± 0.01 e.

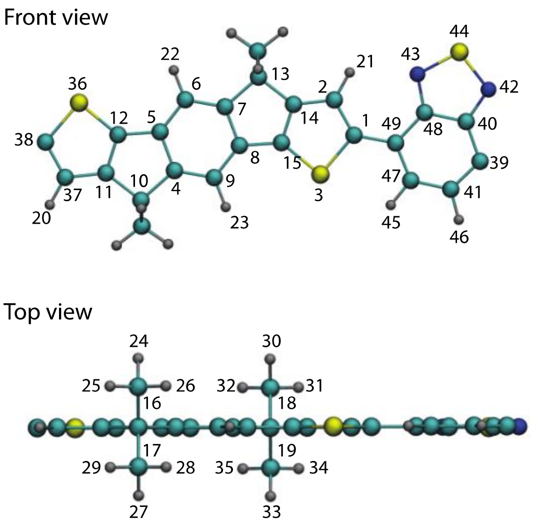

Figure S2. Atom labels as used for force field parameterisation of one IDT-BT repeat unit (RU).

Table S1. IDT-BT repeat unit atomic charges as calculated by CHELPG method. Note that the total charge of each repeat unit is set to zero by redistribution of the total excess charge calculated by CHELPG.

Atom	CHELPG	RU-FF	Atom	CHELPG	RU-FF	Atom	CHELPG	RU-FF
1	0.0271	0.027	18	–0.1733	–0.173	35	0.0484	0.048
2	–0.1135	–0.113	19	–0.1733	–0.173	36	–0.1246	–0.124
3	–0.1202	–0.12	20	0.0721	0.072	37	–0.0898	–0.089
4	–0.153	–0.153	21	0.0692	0.069	38	0.0154	0.015
5	0.1411	0.141	22	0.1694	0.169	39	–0.0258	–0.025
6	–0.2122	–0.212	23	0.1742	0.174	40	0.2328	0.233
7	–0.1363	–0.136	24	0.0072	0.007	41	–0.1263	–0.126
8	0.1274	0.127	25	0.0422	0.042	42	–0.3157	–0.315
9	–0.2110	–0.21	26	0.0146	0.014	43	–0.3115	–0.311
10	0.4616	0.461	27	0.0072	0.007	44	0.2580	0.258
11	–0.1156	–0.115	28	0.0146	0.014	45	0.1437	0.144
12	–0.0012	–0.001	29	0.0422	0.042	46	0.1384	0.138
13	0.4413	0.441	30	0.0097	0.01	47	0.2141	0.214
14	–0.0813	–0.081	31	0.0484	0.048	48	–0.1584	–0.158
15	–0.0100	–0.009	32	0.0204	0.02	49	–0.0039	–0.003
16	–0.1591	–0.159	33	0.0098	0.01	**SUM**	**–0.0040**	**0.000**
17	–0.1591	–0.159	34	0.0204	0.02			

We performed some tests to elaborate on the effect of polymer conformation on the atomic charge distribution. We calculated the Mulliken atomic charges for IDT-BT minimal model at different IDT-BT torsional angles (i.e., $-90° < \phi_{IDT\text{-}BT} < +90°$) and calculated the change in the atomic changes around that torsion, see Figure S3.

We identified the atoms whose atomic charge C_i was most affected by the torsion (see Figure S3, the atoms marked by red). The standard deviation of the charge of each atom can be computed as $STD_i = \left(\langle C_i^2 \rangle_T - \langle C_i \rangle_T^{2}\right)^{0.5}$ with the symbol $\langle\ \rangle_T$ denoting thermal average at 300 K over the Boltzmann distribution of torsional angles. Table S2 shows STD_i for the atoms marked in red in Figure S3-a. As shown, there is only one $STD_i > 0.01$ e and such deviation in atomic charge, considering the precision of classical MD in electrostatic potential calculations, is negligible.

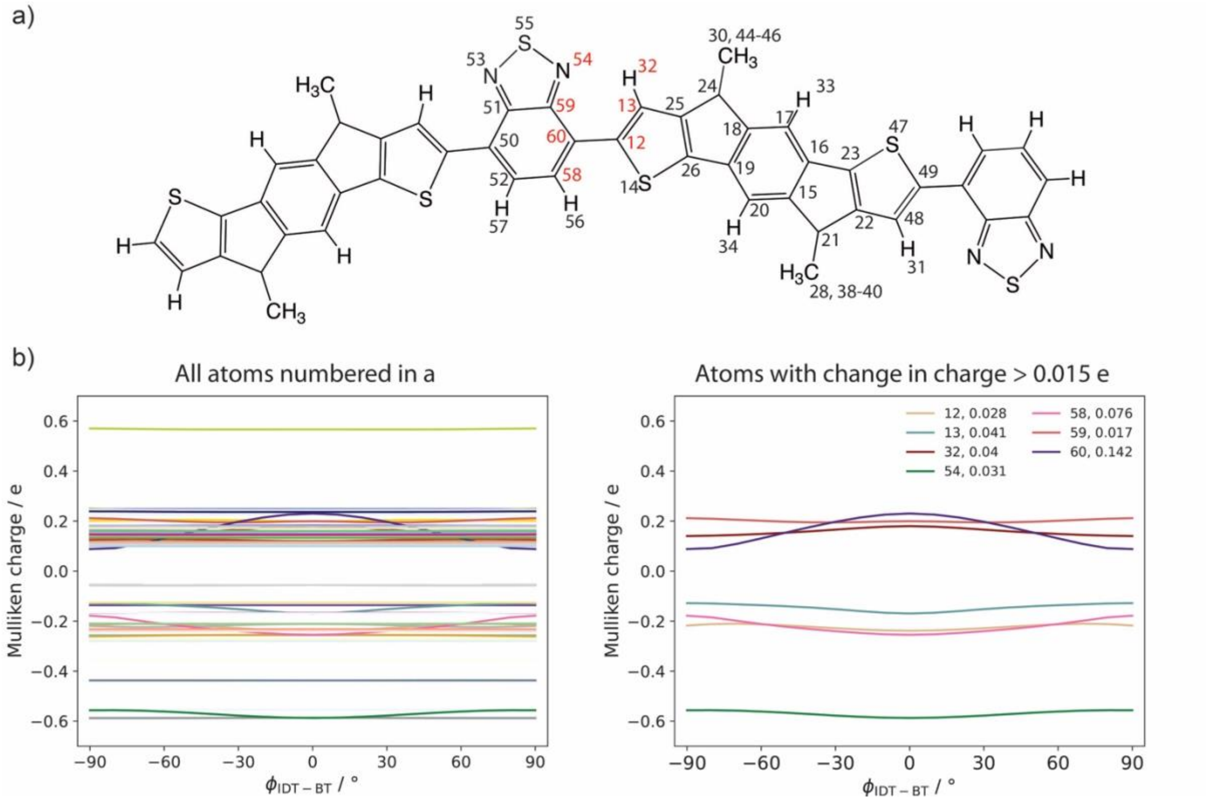

Figure S3. a) the structure of IDT-BT minimal model and the labelling for the atoms around the torsion (the ones with the maximum change in atomic charges > 0.015 e as a result of torsion are highlighted in red). b) The change in Mulliken atomic charges of all atoms labelled in (a), left, and the graph showing only the atoms with change in atomic charges > 0.015 e (right, the legend shows the atom number and the maximum change in charge as a result of torsion).

Table S2. Standard deviation of charge distribution during torsional angle scan (from -90 to +90 °) for atoms most affected by the change in chain conformation, as recognised in Figure R3.

Atom No.	12	13	32	54	58	59	60
STD / e	0.0044	0.0070	0.0059	0.0037	0.0065	0.0016	0.0153

S1.2. Bonded parameters

S1.2.1. Bonds and angles

Harmonic potentials in the forms of equations SE1 and SE2 were used for implementing bond and angle interactions, respectively, in the force field.

$$V_b(r_{ij}) = \frac{1}{2} k_{ij}^{b} (r_{ij}-b_{ij})^2 \qquad \text{(SE1)}$$

$$V_a(\theta_{ijk}) = \frac{1}{2} k_{ijk}^{\theta} (\theta_{ijk} - \theta_{ijk}^{0})^2 \qquad \text{(SE2)}$$

where V_b and V_a are bond and angle potentials, k^b and K^θ are the force constants representing the stiffness of the bond and angle, and b_{ij} and θ_{ijk} are the bond and angle equilibrium values taken from DFT-optimised repeat unit for each combination of bonded atoms.

The stiffness of all bonds (k_{ij}^{b}) and angles (k_{ijk}^{θ}) were set to 320,000.0 kJ/mol/nm^2 and 500.0 kJ/mol/rad^2.

We generated force field for IDT-BT minimal model based on the three major widely used FF parameters (i.e., OPLS, CHARMM, and GAFF) for polymers. Then, we performed MD simulations in vacuum for one chain at 300 K by using our and the other three FFs and compared the bond length distribution calculated for 2000 samples taken over 200 ps of MD simulation under NVT condition. Note that we used the same run file (i.e., the same MD

settings) for all simulations and for our analysis, we selected the bonds with the *stiffest* and *softest* force constants (shown in orange and blue, respectively) in all three FFs, see Table S3, to capture the maximum possible differences in bond length distribution which these force constants could generate (e.g., it includes sp2C-sp3C and sp2C-sp2C bonds).

An immediate observation from Table S3 is that different FFs give a rather wide range of force constants for a similar bond, i.e., typically about 30% difference. Second, the maximum difference in the standard deviation from the average bond lengths for all FFs (including ours) is below 0.005 Å.

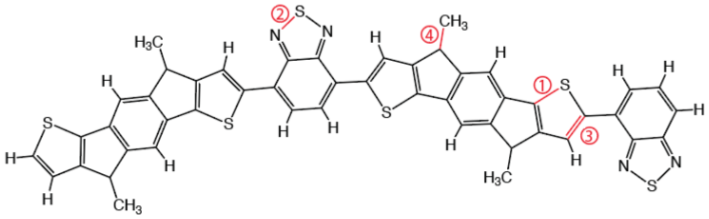

Table S3. Equilibrium bond lengths, the force constants, and the standard deviation from average (STD) of the bond length distribution obtained from the MD simulation (shown in bold) for the four selected bonds shown above. The minimum and maximum bond force constants for the molecule in each force field are marked with *blue* and *orange*, respectively.

		Bond 1	Bond 2	Bond 3	Bond 4
Bond length [nm] / Force constant [kJ mol^{-1} nm^{-2}]<br><br>STD of Bond length in MD [nm]	OPLS	0.1760 / *209200*<br>**0.0031**	0.1730 / *209200*<br>**0.0031**	0.1454 / *402500*<br>**0.0026**	0.1529 / 224262<br>**0.0031**
	CHARMM	0.1730 / 251040<br>**0.0030**	0.1700 / 225936<br>**0.0029**	0.1380 / *334720*<br>**0.0024**	0.1528 / *186188*<br>**0.0032**
	GAFF	0.1756 / *222420*<br>**0.0028**	0.1626 / 301080<br>**0.0031**	0.1373 / *419150*<br>**0.0024**	0.1096 / 276650<br>**0.0030**
	Our FF	0.1749 / 320000<br>**0.0028**	0.1645 / 320000<br>**0.0027**	0.1372 / 320000<br>**0.0025**	0.1545 / 320000<br>**0.0027**

S1.2.2. Intra-fragment torsional potentials

The torsional potentials for internal dihedral angles (inside the repeat unit) were implemented in the form of a Ryckaert-Bellemans function (equation SE3) for all dihedral angles around sp2 hybridised heavy atoms. These fragments are flat and rigid due to their (partial) double bond nature. A representative and well-studied example for a flat and sp2 hybridised molecule is the benzene ring; thus, the OPLS constants of Ryckaert-Bellemans function for carbon atoms of benzene molecule was used for these dihedral potentials. It is worth noting that benzene is an ideal molecule if one wants a single torsional potential for conjugated fragments as each bond is 50% double and 50% single. The Ryckaert-Bellemans reads as:

$$V_{rb}(\phi_{ijkl}) = \sum_{n=0}^{5} C_n(\cos(\psi))^n \qquad \text{SE3}$$

where V_{rb} is the torsional potential of the dihedral angle between the planes of ijk and jkl atoms. Note that $\psi=\phi-180$ and C_n are the six constants of the function. The C_n [kJ/mol] values of the C-C-C-C dihedral of the benzene ring from OPLS force field are shown in Table S4.

Table S4. The OPLS force field constants of Ryckaert-Bellemans function for C-C-C-C dihedral angle of benzene.

C_0	C_1	C_2	C_3	C_4	C_5
30.334	0.0	-30.334	0	0	0

For the dihedral angles in which the sp3 hybridised carbons (e.g., the connecting carbon to the sidechain) take part, equation SE4 was used (proper dihedral type 1 in GROMACS). Note

that all equilibrium dihedral angle values (ϕ_s in equation S4) were directly taken from the optimised repeat unit.

$$V_d(\phi_{ijkl}) = k_\phi(1+\cos(\phi-\phi_s)) \qquad \text{SE4}$$

k_ϕ of 10 kJ/mol was used for all dihedrals around the sp3 carbons connecting the backbone to the sidechains. Note that ϕ = 0 corresponds to the cis configuration (i.e., *ijk* and *jkl* on the same side).

S.1.2.3. Inter-fragment torsional potential

Torsional potential (V^{DFT}) of the dihedral angle between fragments inside the repeat unit (e.g., IDT and BT ϕ_{IDT-BT} in the case of IDT-BT as marked with red circles in Figure S4 a) was calculated by DFT (via B3LYP/6-31g*) through a dihedral scan with 10 ° spacing (in total 37 scans from –180° to 180°). Figure S4 b shows the calculated torsional potential as a function of dihedral angle for IDT-BT. It should be noted that V^{DFT} shows the total potential energy of the monomer structure at each of 37 dihedral angle points. Therefore, after implementing this torsional potential correctly as a force field parameter, the total potential energy of the monomer at each dihedral angle as calculated by the force field should match the $V^{DFT}(\phi_{IDT-BT})$. To this end, we used the parametrisation scheme as explained below.

The DFT-optimised structures at each dihedral angle (i.e., –180, –170, …, 170, 180) were obtained and used as the input coordinate file. Then, for each structure, an energy minimisation based on steepest descent algorithm using the generated force field parameters (excluding the targeted inter-fragment torsional potential) with a stiff dihedral restraint (> 50,000 kJ/mol/rad), which ensures that dihedral angle remains reasonably constant (< 1° fluctuations) during minimisation, was performed. The total energy (V^{FF}), excluding the energies of the restrained dihedral, for each structure (in total 37 values) was calculated after energy minimisation.

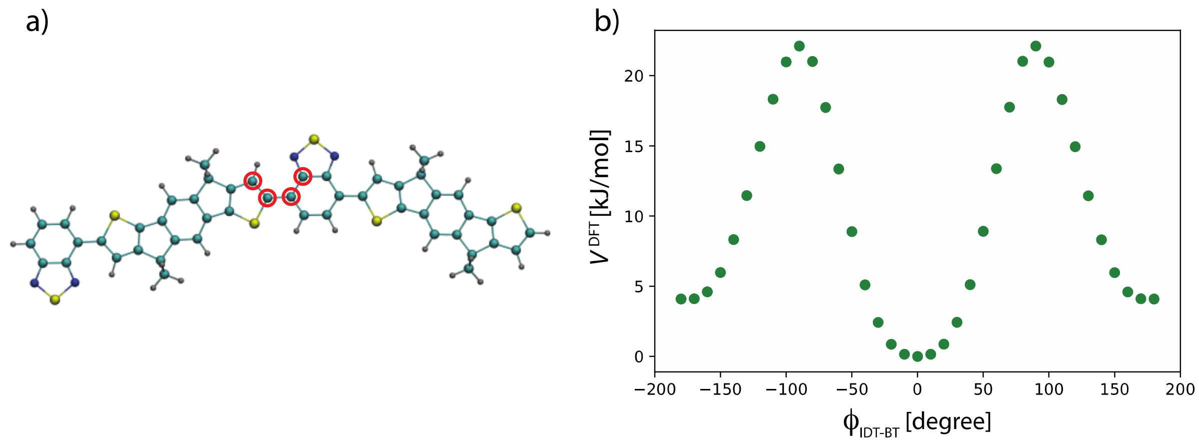

Figure S4. (a) IDT-BT monomer capped with BT (left-end) and IDT (right-end) molecules; the red circle shows the place of rotation for dihedral scan (ϕ_{IDT-BT}). (b) DFT calculated torsional potential by B3LYP/31-6g*.

Figure S5 a shows V^{FF} values for all 37 structures for ϕ_{IDT-BT}. Accordingly, the torsional potential for the force field will be $V^{CORR} = V^{DFT} - V^{FF}$. Figure S5 b shows the correct torsional potential for ϕ_{IDT-BT}, as it is given to the force field in the format of a tabulated potential for MD simulations. The tabulated dihedral file was provided as a three-column table. The first column shows angles (-180 to and including 180, 1-degree spacing is recommended), the second column is the potential value (a cubic spline was fitted on 10-degree spline and the potential for every degree was calculated), and the third column represents the negative value of the first derivates of the potential (i.e., force) as obtained from the cubic spline fit.

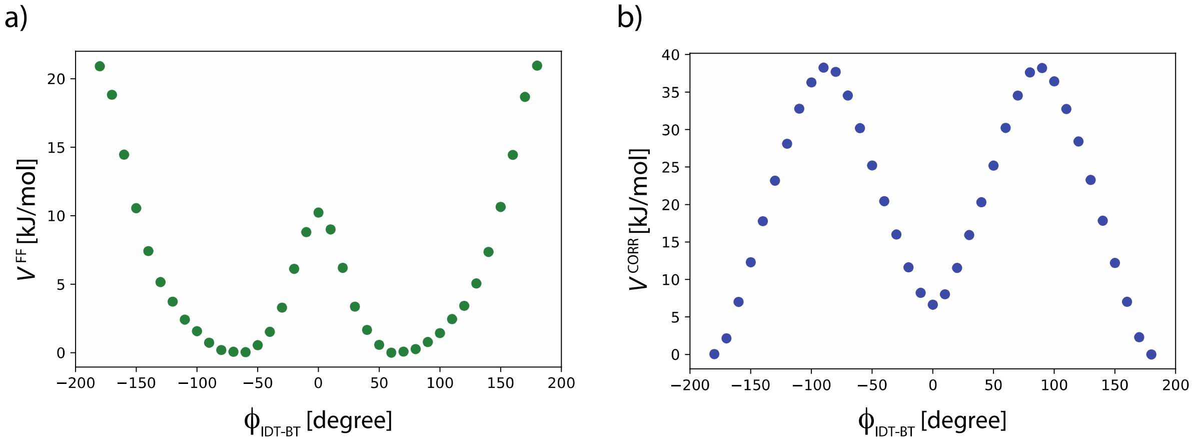

Figure S5. (a) Total energy (excluding the restraint energy imposed to keep ϕ_{IDT-BT} constant) based on the force field (excluding the ϕ_{IDT-BT} torsional potential) for energy minimised structures. (b) Force field torsional potential ($V^{CORR} = V^{DFT} - V^{FF}$).

As a final check, we calculated the total energy of the monomer, but this time the force field torsional potential (V^{CORR}) was also included. Figure S6 shows the comparison between the total energy (excluding the dihedral restraint energy) from force field (V^{tot}) and the DFT calculated torsional potential (V^{DFT}) for all five polymers. As shown and expected, the total potential energy of the repeat-unit after imposing the implemented torsional potential (V^{CORR}) matches the DFT calculated values (V^{DFT}). Note that the small differences in V^{tot} and V^{DFT} is due to the small drift in the fixed torsion during minimisation.

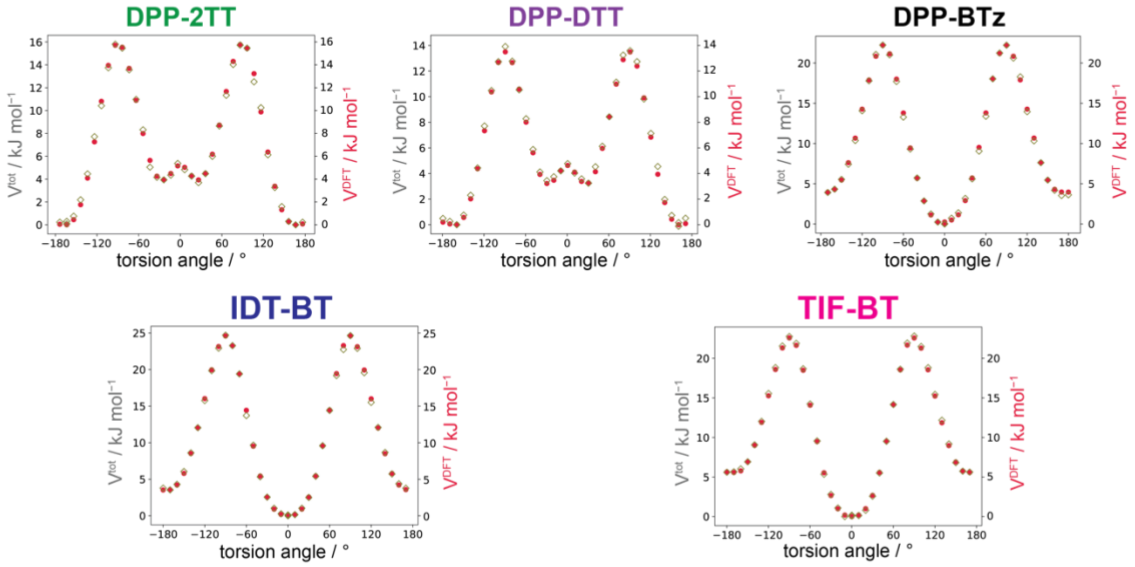

Figure S6. Comparison between DFT calculated (V^{DFT}) and force field calculated (V^{tot}) total potential energy at each dihedral angle of ϕ_{IDT-BT}.

S1.3. Sidechain attachment procedure

The optimised RU structure (coordinates) and force field parameters have been obtained so far. The next step is to attach side chains to the repeat unit and add the force field parameters accordingly. As earlier mentioned, OPLS force field parameters were used for sidechains. However, the interactions between the backbone and the sidechain at the connection points should be treated correctly.

First, the sidechains are assumed to have a total zero net charge. Each methylene ($-CH_2-$) and the end methyl (CH_3) group for each sidechain are parametrised by the united atom parameters of OPLS. Therefore, the total charge of each united atom (and accordingly, the charge of each sidechain) is zero. However, to attach a side chain to each of four methyl groups in the RU, one hydrogen atom of the methyl group should be removed, and its charge

should be redistributed on the remaining two hydrogen atoms, see Figure S7 for IDT-BT repeat unit. In this way, the total charge of the repeat unit with sidechain (RU-SC) will remain zero.

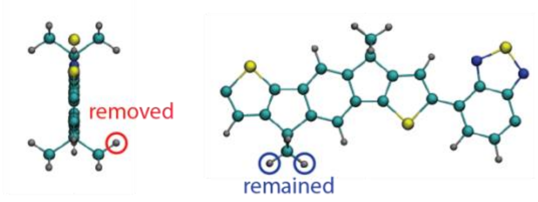

Figure S7. One hydrogen of each methyl group attached to IDT is removed (red circle) and its charge is redistributed on the two remaining hydrogens (blue circles).

The bonded potentials defined around the connection points also need a reasonable treatment. Figure S8 shows the RU-SC structure for IDT-BT. The atom labelling around one of the two connection points are shown. The sp2 hybridised carbon of IDT are shown in green numbers, the sp3 carbon and hydrogen atoms are represented by blue numbers, and the sidechain united atoms are marked with red numbers.

After removing one hydrogen of each methyl group, all the bonded parameters in which this hydrogen was involved were removed from the force field. Then the new bonded potentials were added to the force field as explained here. Based on the atom labelling shown in Figure S8, the bond potential for 16-24 bond was taken from OPLS and added to the force field. Also, all angle potentials for any newly formed angle in which any sidechain united atom exists (e.g., 51-24-16, 24-16-26, 24-16-25, 24-16-10, etc.) were taken from OPLS and added to the force field. In the same way, the potential of all the newly formed dihedrals in which any sidechain united atom exists (e.g., 51-24-16-26, 24-16-10-17, etc.) excluding the ones where sp2 hybridised carbons of the IDT also exist (i.e., 24-16-10-11, and 24-16-10-4) were taken from OPLS and added to the force field. Excluding the two dihedral potentials for each sidechain in which one sidechain united atom (e.g., 24) and one sp2 hybridised carbon atom (e.g., 4 and 11) take part to avoid any distortion in the flatness of the conjugated fragment (IDT) by imposing additional dihedral potentials on it.

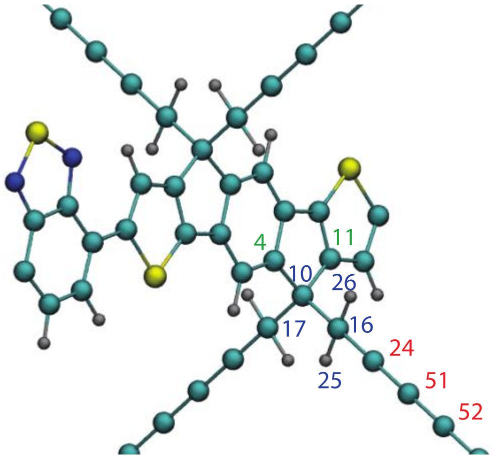

Figure S8. Atom labelling of the RU-SC (repeat unit with side chain). Green, blue, and red coloured numbers represent sp2 carbons, non-sp2 hybridised atoms, and sidechain united atoms, respectively.

S1.4. Polymer models

For all structures as shown in Figure S9, a polymer with *DP* (degree of polymerisation) = 10 was made. Polymer force fields are simply made by recycling the force field of each RU-SC unit. Note that all polymers were capped with two H atoms (with zero charge) at both ends and improper dihedral potentials were used to keep the hydrogen coplanar with the backbone of the polymer.

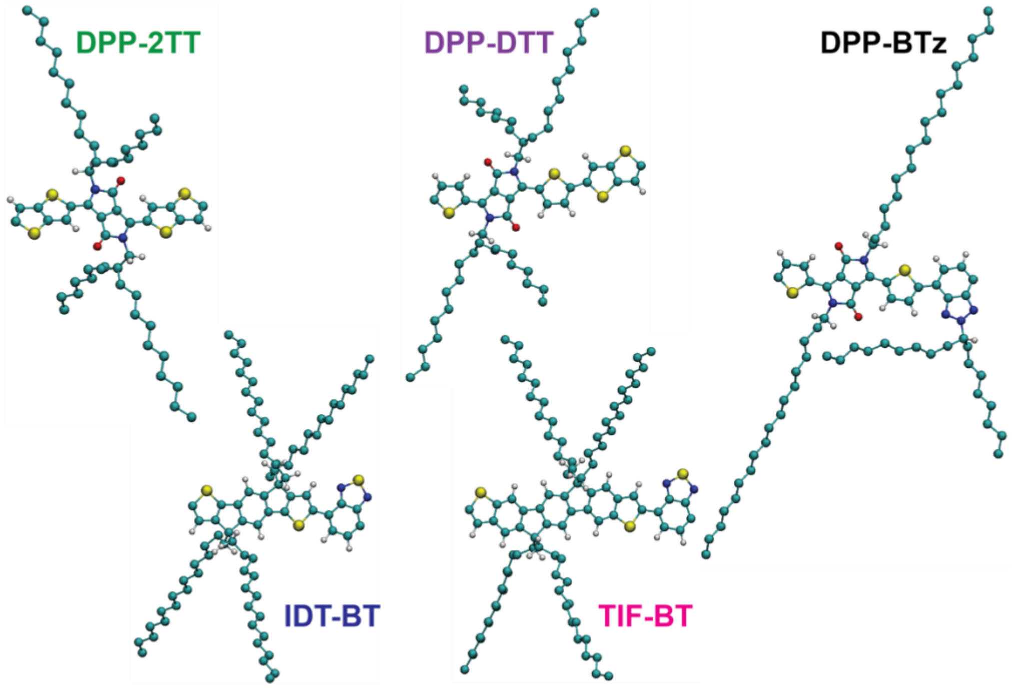

Figure S9. Repeat units of all five SCPs with sidechains attached. The sidechain structures are taken from [S1-S5] for DPP-2TT, DPP-DTT, DPP-BTz, IDT-BT, and TIF-BT, respectively.

S1.5. Implicit solvent model vs "soup" in representing electrostatic environment

Table S5 shows that the calculated values for 30 chain conformations predicted by the "soup" model in different conditions: (i) isolated chain with no surrounding charges, (ii) the surrounding charges as predicted by the soup method, and surrounded by implicit solvents, i.e., (iii) benzene, and (iv) chloroform. Looking at the standard deviation of the HOMO energies (the lowest row of the table), one can clearly observe that the surrounding charges obtained from "soup" method show considerably different HOMO energy distributions compared to the ones predicted by the two implicit solvents. In fact, the values obtained from the implicit solvents are very close to the isolated chains.

Table S5. The average and standard deviation from the average of HOMO energies for IDT-BT and DPP-BTz polymers calculated for isolated chain, "soup" model, the chain in benzene, and chloroform implicit solvents.

Polymer	IDT-BT				DPP-BTz			
Calculation type	Isolated	Soup	Benzene	Chloroform	Isolated	Soup	Benzene	Chloroform
HOMO E (avg) [eV]	-4.71	-4.71	-4.79	-4.84	-4.80	-4.68	-4.93	-5.00
HOMO E (std) [eV]	0.0478	0.0717	0.0467	0.0462	0.0464	0.116	0.0427	0.0414

We also calculated the correlation between isolated chain HOMO energy and HOMO energy in "soup", implicit benzene, and implicit chloroform for the 30 chain conformations in Figure S10. It is clear to see that inclusion of implicit solvent, in each case, has the effect of uniformly shifting the HOMO energies relative to the isolated chain, and as such does not capture the electronic disorder induced by electrostatic interaction between the chain and its surroundings.

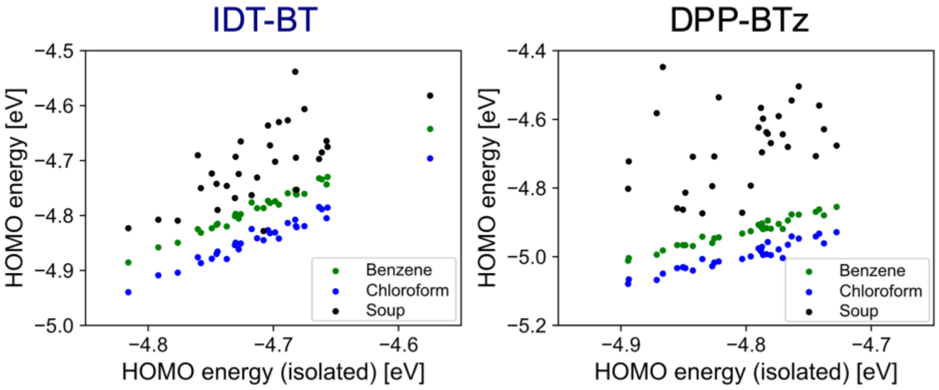

Figure S10. Correlation between HOMO energies calculated for 30 isolated chain conformations of IDT-BT (left) and DPP-BTz (right) and those calculated for the same conformations using either implicit solvent (benzene – green, chloroform – blue), or point charge distributions from the soup model (black).

S2. Equilibration and analyses details

S2.1. T_g and polymer dynamics evaluation from bulk simulations

We performed a temperature-sweep simulation in which the temperature decreased stepwise (20 K steps) from 1000 to 200 K with a total 5 K/ns cooling rate. Then, we used a bilinear fit approach to estimate the T_g of SCPs (see Figure S11). The density vs temperature dependence has a linear relationship below the T_g, which corresponds to the glassy coefficient of thermal expansion, and above the T_g, which corresponds to rubbery coefficient of thermal expansion. The intersection of the bilinear fit in these two regions corresponds to T_g.

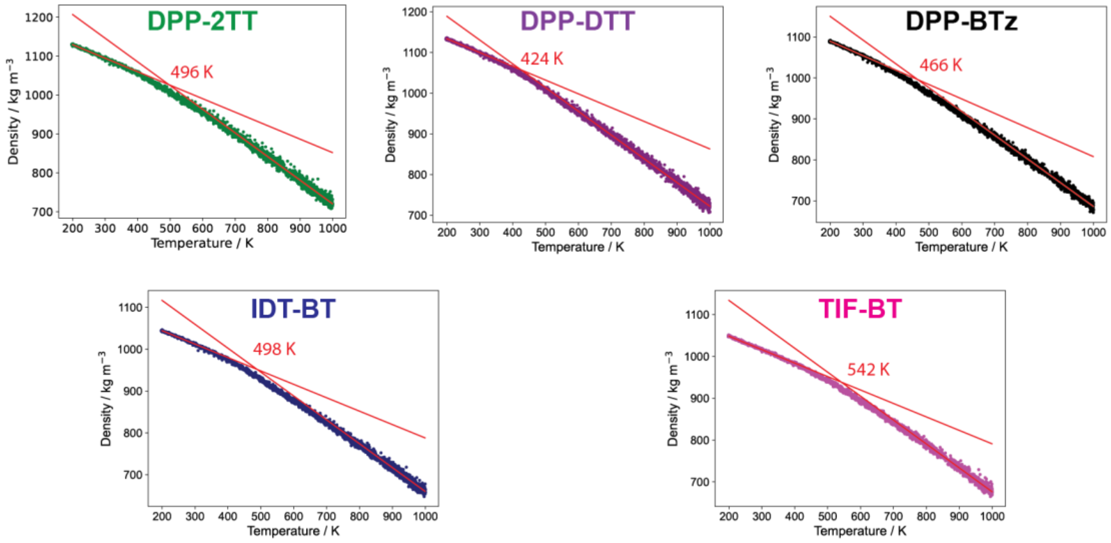

Figure S11. Density-temperature curves for all polymer models. The intersection of fitted lines on the data well-above (1000-900 K) and well-below (200-300 K) of the transition is used to estimate T_g. The cooling rate for all simulations is 5 K/ns. The data in range of 1000-900 K and 300-200 K was used for rubbery and glassy region fitting.

Assessing polymer chain mobility directly through mean squared displacement (*MSD*) is a more robust approach compared to relying on the abovementioned T_g evaluation method to find rubbery and glassy states due to the uncertainties associated with extremely high simulation cooling/heating rates, different fitting procedures, considerably wide range of transition region, etc. of the latter approach. As an example, we calculated the T_g of all five SCPs from the exact same simulations but with slightly different fitting criterion, i.e., 1000-800 K and 400-200 K instead of 1000-900 K and 300-200 K. The estimated T_g value shifted about ± 20-100 K. Thus, we calculated the diffusion coefficient *D* of all atoms during the temperature-sweep simulation for box of polymers (50 10-mer chains), dimers (150 2-mer molecules) and

monomers (300 1-mer molecules), based on their MSD values, and the results are shown in Figure S12. Separate transition temperature regions for 1-mer, 2-mer, and 10-mer models can be seen. Polymers show a more gradual transition in *D* between 700 and 800 K (depending on the polymer structure); however, 1-mer systems show a more abrupt transition at around 450 K for all structures.

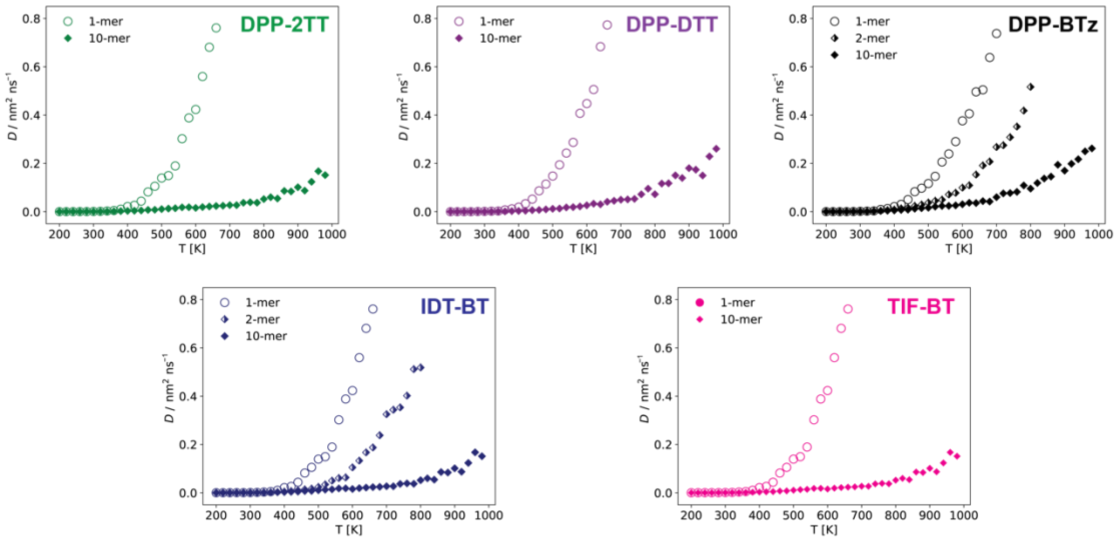

Figure S12. Diffusion coefficient *D* vs temperature graphs for the repeat unit (1-mer), dimer (2-mer) and polymer (10-mer) models.

S2.2. Validation of the models and equilibration schemes

The X-ray scattering pattern of two DPP-based and two BT-based polymers were calculated and compared. As shown in Figure S13a, the main expected microstructural features (i.e., the backbone peaks, marked with ◆, and the broad scattering peak associated with the sidechain, marked with ●) exist for all polymer models. Moreover, the shift in the backbone peaks towards smaller q values, for both DPP- and BT-based polymer models, as the repeat unit size increases is a logical occurrence (the end-to-end size of the DFT-optimised repeat units are also shown in Figure 13a). More importantly, the position of the peaks and the aforementioned shift in the backbone peaks for IDT-BT and TIF-BT match nicely with the experimental data [1] shown in Figure S13b. Unfortunately, to the best of our knowledge, no experimental GIWAXS data has been reported for DPP-2TT and different data reported for DPP-DTT [2-4] show three to four clear backbone peaks at slightly different q values but very consistent with our simulated X-ray pattern.

Figure S13c shows the average end-to-end distance L_e of all polymer models through the equilibration process. As is evident, although the average size and the standard deviation from the average do not considerably change throughout the equilibration, a slightly larger fluctuation and for some cases (e.g., DPP-DTT and DPP-BTz) a very gradual increase in L_e up to 10 annealing cycles can be seen. Therefore, for QC/MD calculations, we only used the samples taken after 20 annealing cycles, as marked with a green highlight in Figure S13c.

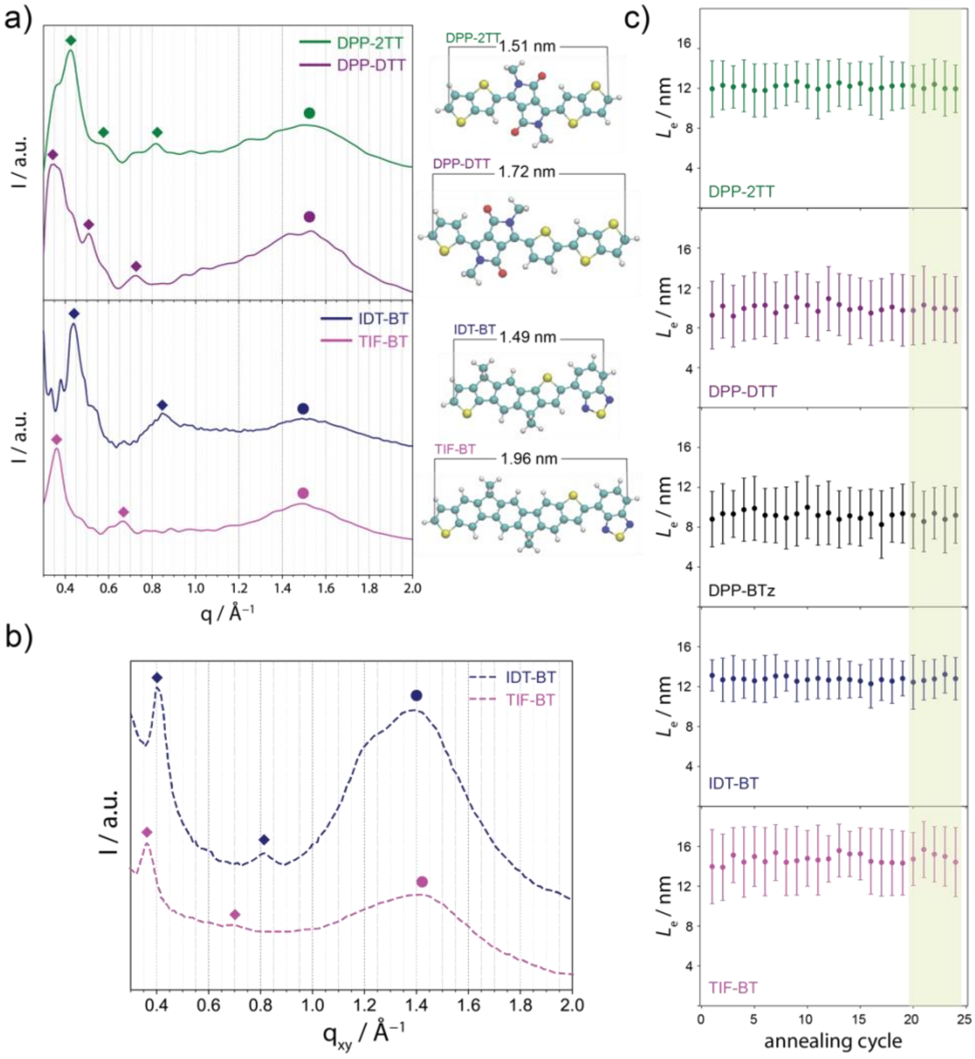

Figure S13. a) Simulated X-ray scattering pattern for DPP-2TT, DPP-DTT, IDT-BT, and TIF-BT polymers. b) GIWAXS linecuts of in-plane direction for IDT-BT and TIF-BT taken from ref [1]. c) Average end-to-end distance L_e (over 50 chains per snapshots) obtained from "melt" equilibration at the end of each annealing cycles. The green-highlighted region (from 20-24) indicates the snapshots taken for QC/MD calculations. Error bars show the standard deviation from the average value.

S2.3. Sampling correlation assessment for "melt" and "soup" methods

We use block averaging to estimate the correlation between the samples obtained from "melt" and "soup" methods for DOS calculations. We considered a full range of block sizes- the largest size is one fourth of the total simulation time. In general, small blocks tend to be highly correlated with neighbouring blocks, whereas blocks longer than important correlation times will only be weakly correlated. The block averaging analysis implicitly detects such correlations. We calculated the standard deviation from average end-to-end distance L_e of polymer chains in each block and plotted them as a function of block size for IDTBT and DPP-2TT polymers in Figure S14, similar results were obtained for other polymers. Note that once the standard deviation is independent of the block size (i.e., reaching the plateau), it reflects essentially independent blocks. As shown, the minimum time between two independent snapshots for "melt" models at 1200 K (which is the well-above T_g temperature we used for all polymers) is about 7.5 ns and for "soup" models at 900 K is about 4 ns. Therefore, we are confident that all 250 samples obtained from "melt" (one every annealing cycle which consists of 10 ns relaxation at 1200 K) and "soup" (one every 4 ns at 900 K) methods are uncorrelated.

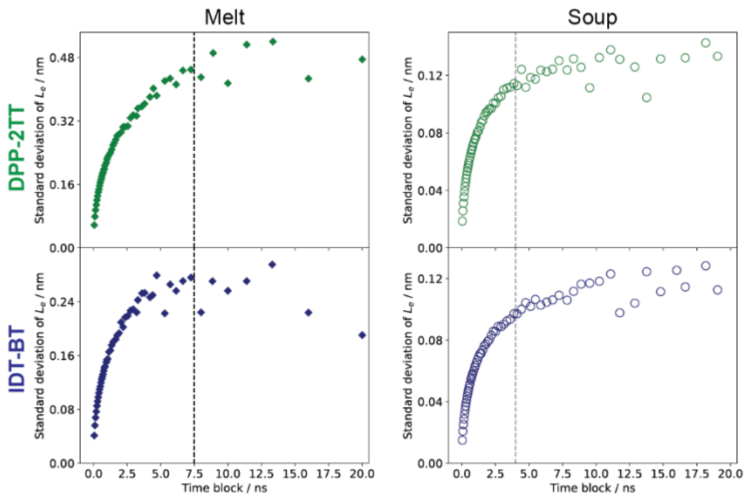

Figure S14. Standard deviation as a function of time block for block averaging analysis. Dashed lines show the time between the closest samples we obtained from “melt” and “soup” simulations.

S2.4. Inter-monomers torsion angle distribution for DPP-based polymers

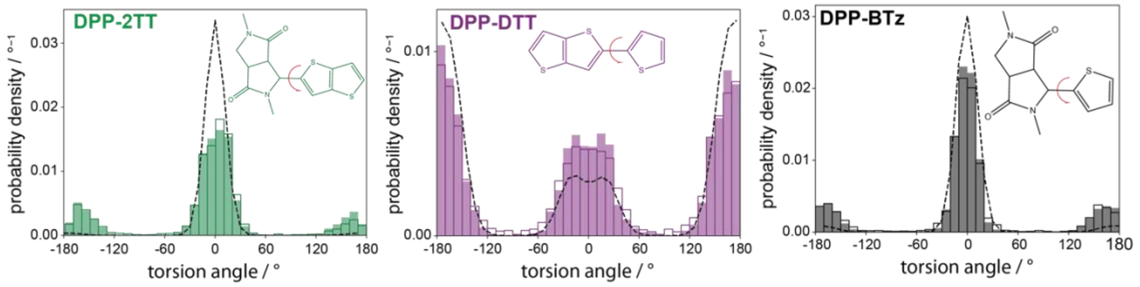

Figure S15. Torsion angle distribution for intra-repeat unit torsions for DPP-based equilibrated polymers obtained from “melt”, filled bars, and “soup”, unfilled bars, methods. The black dashed lines show the corresponding Boltzmann distribution of torsion angles as obtained from DFT scans on the representative repeat-unit molecule.

S2.5. Additional *rdf* and π- π interaction analysis

The like-like rdf of the centre of mass of monomers with highest and lowest HOMO energies in the repeat unit structure of all polymers are calculated and shown in Figure S16. It should be noted that although the “soup” method accurately represents the intra-chain characteristics, it fails to correctly predict the inter-chain morphology for all polymers.

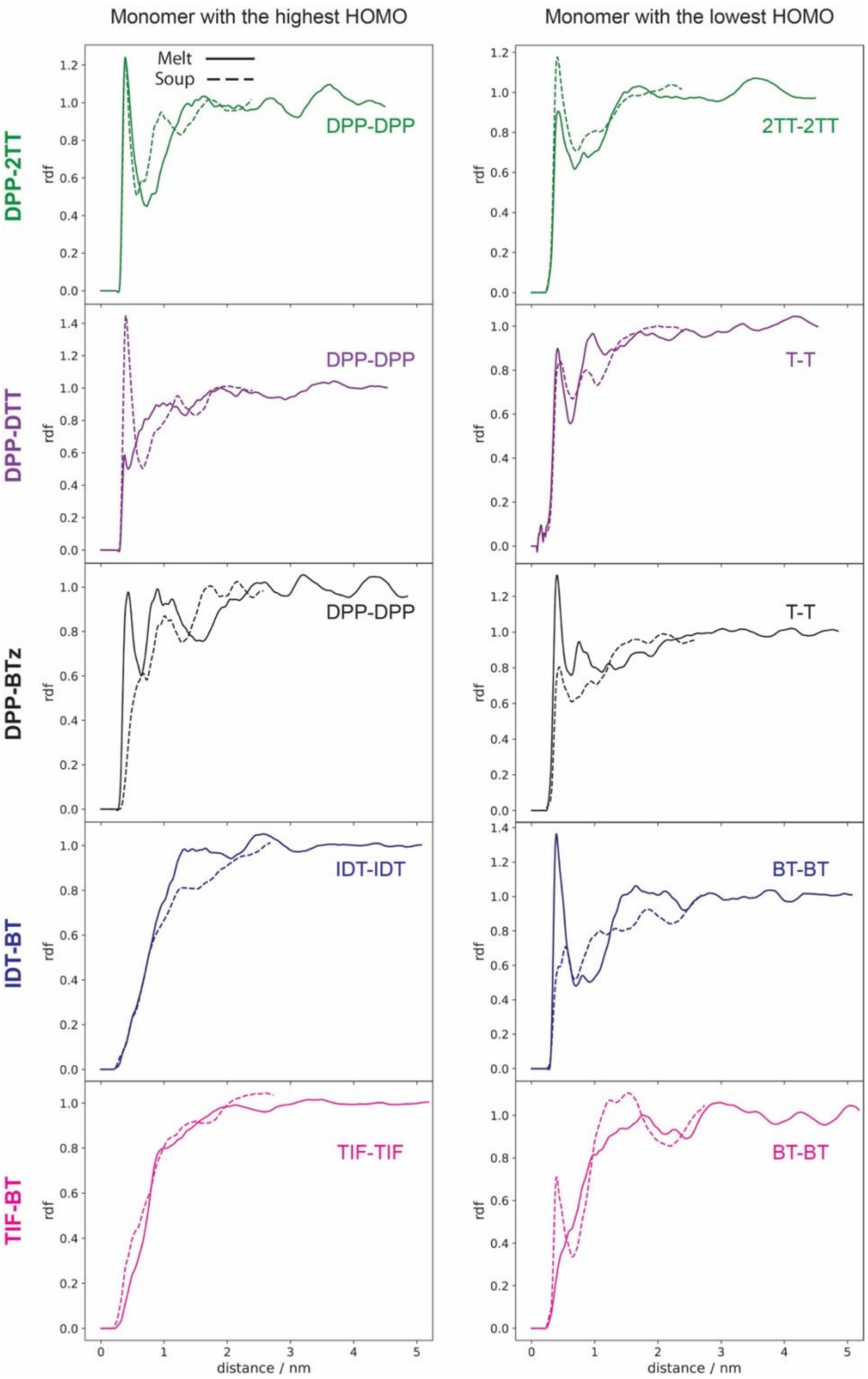

Figure S16. *rdf* of the centre of mass of the monomers with the highest (left) and the lowest (right) HOMO energies as calculated for "soup" and "melt" models.

Table S6 shows the relative number of π-π interacting monomers as estimated by "soup" and "melt" methods. Although there is a strong correlation between the two methods (correlation coefficient >0.8), the "soup" approach generally overestimates the number of π-interacting monomers due to the easier mobility and packing of the repeat units compared to polymer chains.

Table S6. relative number of π-π interacting monomers as estimated by “soup” and “melt” methods.

Polymer	Monomers in π-stacking	π- π stacked / total [%]	
		“melt”	“soup”
DPP-2TT	DPP-DPP	25.6	52
	2TT-2TT	38.6	38.6
DPP-DTT	DPP-DPP	21.6	36.1
	2TT-2TT	17.5	13.2
	T-T	21.8	27.8
DPP-BTz	DPP-DPP	16.2	35.4
	BTz-BTz	17.8	21.2
	T-T	30.4	42.4
IDT-BT	IDT-IDT	< 1	< 1
	BT-BT	7.2	11.8
TIF-BT	TIF-TIF	0.4	0.8
	BT-BT	1.2	0.7

S3. The electrostatic disorder generated by SCP on the surrounding

Band tail gradient and the energy at inflection point of the band tail for all polymers are provided in table S7.

Table S7. Band tail gradient and energy at the inflection point for all polymers as obtained by “melt” and “soup” approaches.

Polymer	Band tail gradient / eV^{-2} $monomer^{-1}$		Energy at inflection point / eV	
	Melt	Soup	Melt	Soup
DPP-2TT	-7.07	-6.86	-4.81	-4.83
DPP-DTT	-5.74	-5.86	-4.74	-4.80
DPP-BTz	-5.34	-5.26	-4.70	-4.69
IDT-BT	-7.04	-5.98	-4.72	-4.69
TIF-BT	-8.50	-7.05	-5.04	-5.06

We constructed a shell around one repeat unit (DFT optimized by B3LYP/6-31g*) and calculated the EP generated by the atoms of the repeat unit on that shell in the following way:

(i) Made a sphere around each atom (500 points equally distanced) with the vdW radius of the atom and removed the points that overlap with other atoms (any points that are within the vdW distance of more than one atom).

(ii) Calculated the EP on each point on the shell and calculated the standard deviation from the average.

Figure S17 shows the structure and EP on the shell points for all SCPs. Table S8 summarises the standard deviation from the average EP on each shell. The relatively larger value for DPP-based polymers is most likely due to the higher electron density on the two oxygens in the structure as one can see the very dark areas around those two oxygen atoms, note that the orientation of the molecule structures and the shells is similar.

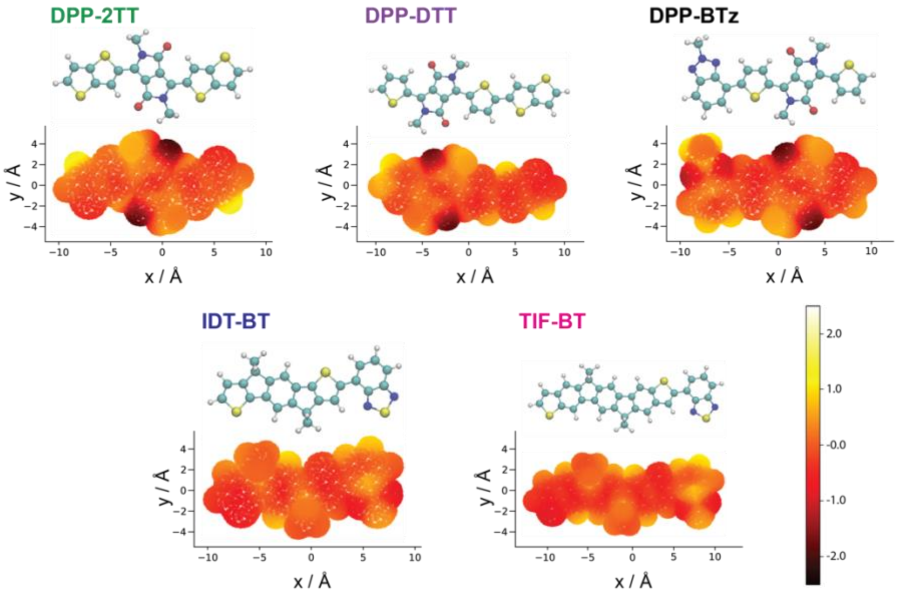

Figure S17. Electrostatic disorder generated by SCPs on the surroundings as characterised by electrostatic potential generated by their repeat units on a shell (with a vdW radius) around them. The colour bar is used to show the magnitude of the electrostatic potential at each point on the shells.

Table S8. Standard deviation (STD) from the average of electrostatic potential on a shell around the repeat unit of SCPs.

	DPP-2TT	DPP-DTT	DPP-BTz	IDT-BT	TIF-BT
STD of EPs / V	0.61	0.57	0.62	0.39	0.42

References:

[1] Andrew Wadsworth et al., Modification of Indacenodithiophene-Based Polymers and Its Impact on Charge Carrier Mobility in Organic Thin-Film Transistors. *J. Am. Chem. Soc.* **2020**, 142, 2, 652–664. https://doi.org/10.1021/jacs.9b09374.

[2] Yuyin Xi et al., Self-assembly of donor–acceptor conjugated polymers induced by miscible ‘poor’ solvents. *Soft Matter* **2019**,15, 1799-1812. https://doi.org/10.1039/C8SM02517G.

[3] Xinran Zhang et al., Molecular Packing of High-Mobility Diketo Pyrrolo-Pyrrole Polymer Semiconductors with Branched Alkyl Side Chains. *J. Am. Chem. Soc*. **2011**, 133, 38, 15073–15084. https://doi.org/10.1021/ja204515s

[4] Seong Won Kim et al., Stretchable Mesh-Patterned Organic Semiconducting Thin Films on Creased Elastomeric Substrates Adv. Funct. Mater. **2021**, 31, 2010870. https://doi.org/10.1002/adfm.202010870
